# The Validity of Conscientiousness Is Overestimated in the Prediction of Job Performance

**DOI:** 10.1371/journal.pone.0141468

**Published:** 2015-10-30

**Authors:** Sven Kepes, Michael A. McDaniel

**Affiliations:** Department of Management, School of Business, Virginia Commonwealth University, Richmond, Virginia, United States of America; University of Vienna, AUSTRIA

## Abstract

**Introduction:**

Sensitivity analyses refer to investigations of the degree to which the results of a meta-analysis remain stable when conditions of the data or the analysis change. To the extent that results remain stable, one can refer to them as robust. Sensitivity analyses are rarely conducted in the organizational science literature. Despite conscientiousness being a valued predictor in employment selection, sensitivity analyses have not been conducted with respect to meta-analytic estimates of the correlation (i.e., validity) between conscientiousness and job performance.

**Methods:**

To address this deficiency, we reanalyzed the largest collection of conscientiousness validity data in the personnel selection literature and conducted a variety of sensitivity analyses.

**Results:**

Publication bias analyses demonstrated that the validity of conscientiousness is moderately overestimated (by around 30%; a correlation difference of about .06). The misestimation of the validity appears to be due primarily to suppression of small effects sizes in the journal literature. These inflated validity estimates result in an overestimate of the dollar utility of personnel selection by millions of dollars and should be of considerable concern for organizations.

**Conclusion:**

The fields of management and applied psychology seldom conduct sensitivity analyses. Through the use of sensitivity analyses, this paper documents that the existing literature overestimates the validity of conscientiousness in the prediction of job performance. Our data show that effect sizes from journal articles are largely responsible for this overestimation.

## Introduction

Meta-analytic findings are viewed as a primary means for generating cumulative knowledge and bridging the often lamented gap between research and practice [1–4]. However, concerns regarding meta-analytic results and our cumulative knowledge remain [5–10]. Sensitivity analyses address the degree to which the results of a meta-analysis remain stable when conditions of the data or the analysis change [11]. To the extent that results remain stable, they can be considered robust. Unfortunately, the vast majority of meta-analyses in the organizational sciences fail to conduct sensitivity analyses and do not report the robustness of the meta-analytic findings [12] despite the fact that scientific organizations such as the *American Psychological Association* [13, 14] and the *Cochrane Collaboration* [15] require or recommend such analyses.

Sensitivity analyses in meta-analytic studies include publication bias and outliers analyses. Publication bias occurs to the extent that research findings on a particular relation that are available are not representative of all research findings on that relation of interest [6, 16]. Although publication bias analyses are rare in the organizational sciences, such analyses are much more common in other disciplines. For example, van Lent, Overbeke, and Out examined the role of review processes in the publication of drug trials in medical journals [17]. Kicinski examined publication bias in several meta-analyses in four major medical journals [18]. Publication bias has also been addressed in animal research [19, 20]. In both the medical sciences [21–23] and the social sciences [24], publication bias appears to be primarily driven by authors who do not submit null or otherwise undesirable findings [16, 25]. These authors are likely responding to journal policies that discourage the publication of research with non-significant findings as well as replications that can enable the evaluation of the credibility of previous research findings [10, 26]. In addition to publication bias, outliers can have a noticeable effect on meta-analytic results [27, 28]. Unfortunately, although outlier analyses are a type of sensitivity analysis [11], only around 3% of all meta-analyses in the organizational sciences report assessments of outliers [29].

Our analysis addresses the personality trait, conscientiousness. It is considered one of the “Big 5,” a term that refers to five broad dimensions that succinctly describe human personality [30]. Shaffer and Postlethwaite [31] conducted the most comprehensive meta-analysis to date in which they assessed the correlation (i.e., validity) between conscientiousness and job performance (*k* = 113). Of the Big 5, conscientiousness was found to have the largest magnitude validity (the observed validity range for conscientiousness was .13 to .20 [31]). The authors found that the other Big 5 personality traits had observed mean validities that were less meaningful from a practical perspective in a selection context (i.e., where job performance is the criterion). A concern with the Shaffer and Postlethwaite study is that they concluded that the validity estimates for conscientiousness are not affected by publication bias [31]. However, they did not perform any sensitivity analysis. This paper applies sensitivity analyses, specifically publication bias and outlier analyses, to evaluate the robustness of their conclusions. To facilitate this task, we replicated their approach and crossed the frame-of-reference variable with all other moderators, which allowed us to reduce moderator-induced heterogeneity and to assess whether the influence of outliers and/or publication bias varied across sub-distributions.

## Methods

### Data source

We used data from Shaffer and Postlethwaite that included 113 correlation coefficients [31]. Unless otherwise noted, our sensitivity analyses were conducted using *Comprehensive Meta-Analysis* (CMA, version 2.0 [32]) and follow the recommendations of Greenhouse and Iyengar [11] and Kepes et al. [33]. Given that CMA is based on the Hedges and Olkin [34] tradition of meta-analysis, our results differed slightly from the psychometric meta-analysis method [35] used by Shaffer and Postlethwaite [36]. We note that the reliability coefficients of the personality scales in this data set are between .79 and .87 (based on the coefficients from the data set for measures that reported at least three reliability coefficients).

### Analysis approach

To facilitate understanding of our analysis for those in varying disciplines, we use the term “distribution” to refer to a set of effect sizes. When the effect sizes are sub-divided into smaller groups based on their values on one or more moderator variables, we refer to the subsets of effect sizes as “sub-distributions.” Consistent with the personnel selection literature, we use the term “validity” to describe the correlation between one measure, in this case a self-report assessment of conscientiousness, and a measure of job performance.

First, we derived random-effects (RE) meta-analytic estimates. Second, we conducted one-sample removed analyses to examine the influence of each individual sample on the meta-analytic results [37]. Next, we performed publication bias analyses using multiple methods to triangulate the effect size estimate [38] and to identify the possible range of point estimates (i.e., mean correlations) rather than relying on a single estimate [33]. We used contour-enhanced funnel plots [39], the trim and fill analysis with the *L* estimator [40], selection models [41], and cumulative meta-analysis by precision [37] to perform our publication bias analyses. A modified confunnel command in *Stata* was used to create the contour-enhanced funnel plots [33]. A priori selection models were conducted in *R* [42] with the *p*-value cut-points to model moderate and severe instances of publication bias suggested by Vevea and Woods [41]. In addition, using *R*, we also ran tests of excess significance (P-TES; [43, 44]), PET-PEESE (precision-effect test, precision effect estimate with standard error) analyses [45], whereby PET is Stanley and Doucouliagos’ (formula 6 [45]) modified version of Egger’s test of the intercept [46], and *p*-uniform analyses [47]. P-TES estimates the probability of the obtained results given the statistical power of the primary studies. Thus, contrary to the other analyses, P-TES does not provide an effect size estimate that is adjusted for publication bias. When estimating power for the primary studies, we used the random-effects mean from the distribution as the estimate of the population correlation (*ρ*) and set the significance level at .05. A set of effects with a probability of less than .1 is typically considered to lack credibility [44]. Finally, we used Viechtbauer and Cheung’s outlier and influence diagnostics to identify potential outliers [48]. This procedure was conducted in *R*; it includes seven ‘leave-one-out’ diagnostic measures specifically adapted or developed for the meta-analytic context that examine the influence of each individual study. Viechtbauer [49] included descriptions of these diagnostics and the criteria for determining which study may be considered to be an outlier. We ran all of our analyses with and without the identified outlier(s). As recommended, our results are presented with and without outliers, and we only assess the presence of bias in distributions consisting of at least 10 samples because conclusions from smaller distributions are questionable due to the lack of statistical power and second-order sampling error [33, 35, 50].

Each sensitivity analysis has some limitations, which is why we ran multiple analyses and sought convergence across methods. Next, we address strengths and weakness of trim and fill due to issues raised by reviewers. A *PubMed* search of the words “trim and fill” (or “trim & fill”) from 1999, the date of the dissertation that introduced the method, through 2014 yielded 142 citations. A search of *ProQuest* dissertations yielded 187 citations. We offer that this indicates that the method has many adherents. The primary weakness of the method is that the results can be inaccurate in the presence of heterogeneity (i.e., variance not due to random sampling error) [40, 51, 52]. Thus, in our analyses, the credibility of the trim and fill results is strongest in those sub-distributions in which we control for moderators (and thus control to some degree for heterogeneity) [33]. Regarding the influence of heterogeneity on PET-PEESE, Moreno et al. conducted a comprehensive simulation study that included variants of Egger’s test of the intercept [53]. Two of these variants (fixed-effects model and fixed-effects variance model) correspond to the two components of PET-PEESE. They concluded that these variants can be inappropriate in very heterogeneous settings [53]. Similarly, as noted in the description of *p*-uniform [47], this method overestimates the mean effect as heterogeneity increases. To the extent that our data set has heterogeneity, *p*-uniform, and maybe also PET-PEESE, could be inappropriate. However, because both methods are relatively new, we argue that it is informative to apply them to our data set to see the extent to which their results converge with the results of the other, more established methods.

Finally, we note that some analyses use Fisher’s *z* transformed Pearson’s correlation coefficients (i.e., *r*). The transformation is used in some statistical methods, in part, because it makes the sampling distribution symmetrical. Given the relatively small magnitude of our correlations, the Fisher *z* coefficients and the untransformed correlation coefficients were nearly identical. Still, in the interest of making our analyses clear and our results fully replicable, we detail which statistical methods transformed correlation coefficients into Fisher *z*. The issue is whether the statistical method uses Fisher *z* transformed correlation coefficients in calculations. All methods that did use Fisher *z* coefficients in calculation used a back transformation of the results into untransformed correlation coefficients. The meta-analyses that yielded the random effect mean, the confidence interval for the mean, the *Q* test, the *I* ^*2*^ statistic, and the tau estimate were conducted with CMA, which uses Fisher *z* coefficients in calculations. Although CMA did not provide the prediction interval, we calculated it using output from CMA, which again does calculations using Fisher *z* correlations. Likewise, the one sample removed analyses, and the trim and fill analyses, were conducted using CMA and were thus based on Fisher *z* coefficients. The selection models use Fisher *z* transformed correlations as well. *P*-uniform was also conducted on Fisher *z* coefficients. The PET-PEESE and outlier analyses were conducted using untransformed correlation coefficients. We emphasize that for all results, the coefficients are in the metric of untransformed correlation coefficients and thus can be compared.

### Decision rules for determining the range of the mean estimates and the magnitude of bias

We relied on decision rules offered in Kepes et al. in determining the range of mean validity estimates and the magnitude of publication bias [33]. These decision rules are summarized here. First, we estimated the highest validity defined as the RE meta-analytic mean (${\overset{-}{r}}_{o_{RE}}$). Next, we performed several sensitivity analyses, including the one sample removed analysis (osr), the trim and fill analysis (t&f ${\overset{-}{r}}_{o}$), and selection models with moderate (sm_m_ ${\overset{-}{r}}_{o}$) and severe (sm_s_ ${\overset{-}{r}}_{o})$ assumptions of publication bias to derive additional mean validity estimates. We also conducted P-TES, PET-PEESE, and *p*-uniform analyses. We defined the highest validity estimate as the highest value from any analysis that provided an adjusted effect size estimate (${\overset{-}{r}}_{o_{RE}}$, osr, ${\overset{-}{r}}_{o_{FE}}$, t&f ${\overset{-}{r}}_{o}$, sm_m_ ${\overset{-}{r}}_{o}$, sm_s_ ${\overset{-}{r}}_{o}$, and PET-PEESE) [33]. We excluded the results from *p*-uniform due to their lack of convergence with the results from the other, more established methods. We note that this is likely due to the heterogeneity of our data [47].

We defined the lowest validity estimate as the smallest value from any of these seven analyses (i.e., ${\overset{-}{r}}_{o_{RE}}$, osr, ${\overset{-}{r}}_{o_{FE}}$, t&f ${\overset{-}{r}}_{o}$, sm_m_ ${\overset{-}{r}}_{o}$, sm_s_ ${\overset{-}{r}}_{o}$, and PET-PEESE). We defined the baseline range estimate (BRE) as the absolute difference between ${\overset{-}{r}}_{o_{RE}}$ and the validity estimate farthest away (either the lowest or highest value). We defined the maximum range estimate (MRE) as the absolute difference between the lowest and the highest value. When calculating the relative difference of the range estimates, we used ${\overset{-}{r}}_{o_{RE}}$, the potentially best mean estimate, as the base (i.e., as 100%). Consistent with Kepes et al., we characterized the magnitude of publication bias as negligible if the relative range (BRE or MRE) was smaller than 20%, as moderate if the relative range (BRE or MRE) was between 20% and 40%, and as large if the relative range (BRE or MRE) was larger than 40%. For the P-TES estimates, we used the decision rules from Francis [44] to determine whether the data were suspect (i.e., a probability of .1 or less is consistent with an inference that the data should be viewed with skepticism). We find the decision rules from Kepes and colleagues reasonable, and note that other researchers have used them [54], and, to date, no critiques of them have been offered. However, readers may choose to adopt other decision rules. We provide the data and results needed to assist the reader in such an effort.

## Results

Using the approach detailed by Viechtbauer and Cheung [48] and the diagnostics and criteria for determining whether a particular study is an outlier described by Viechtbauer [49], we identified one outlier (the correlation coefficient from Lao [55]). We verified that the study was correctly coded (see [55], p. 32, Table 1). The sample is composed of police officers (“State Troopers”). Other research has found lower than typical prediction of law enforcement job performance from measures of general cognitive ability and employment interviews [33, 56]. Hirsh and colleagues speculated that the lower magnitude correlations may be due to the supervisor having limited opportunity to observe the work of the police officer [56]. Police officers typically patrol alone in their police car out of the view of their supervisor. Our results by sub-distributions are presented in Table 1 for all primary samples and in S1 Table contains the results without the one identified outlier.

**Table 1:** Meta-analytic and publication bias results.

Distribution	Meta-analysis								Publication bias analyses									
									Trim and fill				Selection models		Ex. sig.	PET-PEESE		*p*-uniform
	*k*	${\overset{-}{r}}_{o_{RE}}$	95% CI	90% PI	*Q*	*I* ^2^	*τ*	osr	FPS	*ik*	t&f ${\overset{-}{r}}_{o}$	t&f 95% CI	sm_m_ ${\overset{-}{r}}_{o}$	sm_s_ ${\overset{-}{r}}_{o}$	P-TES	PET ${\overset{-}{r}}_{o}$	PEESE ${\overset{-}{r}}_{o}$	(95% CI)
Conscientiousness	113	.16	.14, .18	.03, .29	236.52	52.65	.081	.16, .16; .16	L	22	.13	.10, .15	.14 (.01)	.12 (.01)	.24	.09 (.00)	.13	.19 (.16, .22)
Frame of reference																		
- Non-contextualized	91	.15	.13, .18	.00, .29	210.06	57.15	.088	.15, .16; .15	L	15	.12	.09, .15	.13 (.01)	.09 (.01)	.30	.09 (.01)	.13	.20 (.17, .23)
- Contextualized	22	.19	.16, .22	.16, .22	19.01	.00	.000	.19, .20; .19	L	5	.17	.14, .20	.18 (.01)	.18 (.01)	.30	.13 (.03)	.17	.16 (.10, .22)
Source																		
- Journal articles	67	.19	.16, .21	.06, .31	130.89	49.58	.076	.18, .19; .19	L	18	.14	.12, .17	.17 (.00)	.16 (.00)	.39	.07 (.07)	.07	.16 (.10, .22)
- Non-contextualized	52	.19	.15, .22	.04, .32	113.67	55.13	.085	.18, .19; .19	L	14	.14	.11, .17	.17 (.01)	.15 (.01)	.62	.06 (.10)	.06	.21 (.17, .25)
- Contextualized	15	.19	.14, .23	.12, .25	16.61	15.71	.033	.18, .20; .19	L	2	.17	.13, .21	.18 (.00)	.17 (.00)	.36	.07 (.26)	.07	.17 (.10, .24)
- Non-journal articles	46	.12	.09, .15	-.02, .25	91.68	50.92	.080	.11, .13; .12	L	3	.11	.08, .14	.10 (.01)	n/a	.53	.11 (.02)	.11	.18 (.13, .23)
- Non-contextualized	39	.11	.07, .14	-.04, .24	81.35	53.29	.081	.10, .11; .11		0	.11	.07, .14	.08 (.01)	n/a	.65	.12 (.02)	.11	.19 (.13, .25)
- Contextualized	7	.22	.15, .28	.14, .29	1.71	.00	.000	.21, .23; .21										
Purpose																		
- General purpose	76	.14	.12, .17	-.01, .28	175.89	57.36	.089	.14, .15; .14	L	14	.10	.08, .13	.12 (.01)	.08 (.01)	.32	.08 (.03)	.12	.20 (.16, .24)
- Non-contextualized	69	.14	.11, .17	-.02, .29	170.03	60.01	.093	.14, .15; .14	L	9	.11	.08, .14	.11 (.01)	n/a	.63	.09 (.03)	.12	.21 (.17, .25)
- Contextualized	7	.17	.11, .23	.11, .23	4.08	.00	.000	.15, .20; .17										
- Workplace purpose	37	.19	.17, .22	.12, .26	45.77	21.35	.041	.19, .20; .19	L	9	.17	.14, .20	.18 (.00)	.18 (.00)	.20	.12 (.01)	.16	.18 (.14, .23)
- Non-contextualized	22	.19	.15, .23	.09, .29	31.10	32.48	.054	.18, .20; .19	L	5	.17	.13, .21	.18 (.00)	.17 (.00)	.31	.09 (.08)	.09	.18 (.13, .24)
- Contextualized	15	.20	.16, .24	.16, .24	14.15	1.06	.008	.19, .21; .20	L	2	.19	.15, .23	.19 (.00)	.19 (.00)	.64	.19 (.03)	.20	.18 (.12, .25)
Sample																		
- Incumbents	109	.16	.14, .18	.02, .29	230.04	53.05	.082	.16, .16; .16	L	22	.12	.10, .14	.14 (.01)	.11 (.01)	.40	.09 (.01)	.13	.19 (.16, .22)
- Non-contextualized	88	.15	.12, .17	.00, .29	204.61	57.48	.088	.15, .15; .15	L	13	.12	.09, .15	.13 (.00)	.09 (.01)	.49	.09 (.01)	.12	.20 (.17, .24)
- Contextualized	21	.19	.15, .22	.16, .22	18.54	.00	.000	.18, .20; .19	L	5	.16	.13, .20	.18 (.00)	.17 (.00)	.32	.11 (.06)	.11	.16 (.09, .22)
- Applicants	4	.24	.17, .31	.13, .34	.48	.00	.000	.20, .27; .25										
- Non-contextualized	3	.24	.15, .33	-.06, .50	.45	.00	.000	.20, .27; .26										
- Contextualized	1	.23	.11, .34	n/a	n/a	n/a	n/a	n/a										
Design																		
- Concurrent design	105	.15	.13, .18	.02, .28	221.60	53.07	.082	.15, .16; .16	L	21	.12	.10, .14	.13 (.00)	.11 (.01)	.12	.09 (.01)	.13	.19 (.16, .22)
- Non-contextualized	86	.15	.12, .17	.00, .29	199.03	57.29	.088	.15, .15; .15	L	13	.12	.09, .15	.13 (.01)	.09 (.01)	.47	.09 (.01)	.12	.20 (.17, .24)
- Contextualized	19	.18	.15, .22	.15, .21	17.27	.00	.000	.17, .19; .18	L	3	.17	.13, .21	.17 (.00)	.17 (.00)	.29	.11 (.07)	.11	.15 (.08, .22)
- Predictive design	6	.25	.19, .31	.18, .32	1.18	.00	.000	.24, .26; .25										
- Non-contextualized	4	.26	.18, .33	.15, .36	.79	.00	.000	.24, .28; .26										
- Contextualized	2	.24	.13, .34	n/a	n/a	n/a	n/a	.21, .27; .24										
Scale ^a^																		
- NEO	42	.14	.10, .17	-.01, .28	96.28	57.42	.086	.13, .14; .14	L	9	.09	.06, .13	.12 (.01)	.08 (.01)	.53	.08 (.07)	.08	.19 (.14, .25)
- PCI	13	.24	.19, .28	.20, .28	8.71	.00	.000	.22, .25; .23	L	5	.20	.17, .24	.23 (.00)	.22 (.00)	.28	.21 (.14)	.21	.20 (.14, .27)
- PSI	11	.22	.17, .26	.17, .26	4.32	.00	.000	.21, .22; .22		0	.22	.17, .26	.21 (.00)	.21 (.00)	.89	.24 (.00)	.22	.18 (.10, .25)

*Note*: *k* = number of correlation coefficients in the analyzed distribution. Publication bias analyses were not conducted for distributions with less than *k* = 10; ${\overset{-}{r}}_{o_{RE}}$ = random-effects weighted mean observed correlation; 95% CI = 95% confidence interval; 90% PI = 90% prediction interval; *Q* = weighted sum of squared deviations from the mean; *I* ^2^ = ratio of true heterogeneity to total variation; *τ* = between-sample standard deviation; osr = one-sample removed, including the minimum and maximum effect size and the median weighted mean observed correlation; Trim and fill = trim and fill analysis; FPS = funnel plot side (i.e., side of the funnel plot where samples were imputed; L = left, R = right); *ik* = number of trim and fill imputed samples; t&f ${\overset{-}{r}}_{o}$ = trim and fill adjusted observed mean (the weighted mean of the distribution of the combined observed and the imputed samples); t&f 95% CI = trim and fill adjusted 95% confidence interval; sm_m_ ${\overset{-}{r}}_{o}$ = one-tailed moderate selection model’s adjusted observed mean (and its variance); sm_s_ ${\overset{-}{r}}_{o}$ = one-tailed severe selection model’s adjusted observed mean (and its variance); Ex. sig. = excess significance; PET-PEESE = precision-effect test-precision effect estimate with standard error; PET ${\overset{-}{r}}_{o}$ = PET adjusted observed mean (and its one-tailed *p*-value; PEESE ${\overset{-}{r}}_{o}$ is the adjusted observed mean if PET ${\overset{-}{r}}_{o}$ is significant, the PET ${\overset{-}{r}}_{o}$ is the adjusted observed mean if the *p*-value is not significant [45]); PEESE ${\overset{-}{r}}_{o}$ = PEESE adjusted observed mean; P-TES = the probability of the chi-square test of excess significance; *p*-uniform (95% CI) = the *p*-uniform estimate and its 95% confidence interval; n/a = not applicable (because *k* was too small to conduct these analyses or because the variance component for the selection models indicated that the estimate was nonsensical [33]).

^a^ We only analyzed three scale distributions (i.e., NEO = NEO Personality Inventory, PCI = Personal Characteristics Inventory, and PSI = Personal Style Inventory) because the other distributions were too small to reach definite conclusions regarding the robustness of the meta-analytic mean estimate.

Table 1 contains the results of the conscientiousness analyses conducted for the full distribution and publication bias results are offered for all sub-distributions with at least 10 correlations. The first two columns in Table 1 show the distribution analyzed and the number of samples (*k*) in the distribution. Columns three through nine display the results from the meta-analytic RE model: the mean observed correlation (${\overset{-}{r}}_{o_{RE}}$), the associated 95% confidence interval (95% CI), the associated 90% prediction interval (90% PI), the *Q* statistic, *I* ^2^, *τ*, and the one-sample removed analysis (minimum, maximum, and median mean validity estimates). The next four columns (10 through 13) contain the results from the trim and fill analysis, including the side of the funnel plot where the samples were imputed (FPS; a left-hand side imputation is consistent with an inference of publication resulting from the suppression of small magnitude effect sizes; [33, 40]), the number of imputed samples (*ik*), the trim and fill adjusted observed mean correlation (t&f ${\overset{-}{r}}_{o}$), and the trim and fill adjusted 95% confidence interval (t&f 95% CI). Columns 14 and 15 display the results from the moderate and severe selection models, including their respective adjusted observed estimates for instances of moderate and severe publication bias (sm_m_ ${\overset{-}{r}}_{o}$ and sm_s_ ${\overset{-}{r}}_{o}$) and their respective variance component. Column 16 provides the probability for the test of excess significance (P-TES). We report the probability of the chi-square test as the P-TES value and note that this is a probability of excess significance and is not an effect size. The next two columns, column 17 and 18, display the PET (precision-effect test) and PEESE (precision effect estimate with standard error) adjusted observed mean estimates (i.e., PET ${\overset{-}{r}}_{o}$ and PEESE ${\overset{-}{r}}_{o}$, respectively; the PET ${\overset{-}{r}}_{o}$ column also includes its associated one-tailed *p*-value, which is used to determine whether the PET ${\overset{-}{r}}_{o}$ or the PEESE ${\overset{-}{r}}_{o}$ is the adjusted observed mean for the meta-analytic distribution [45]). The final column contains the *p*-uniform adjusted estimate of the mean effect size and its 95% confidence interval (*p*-uniform [95% CI]).

We note that the P-TES values changed substantially in a few distributions when the sole outlier was dropped, suggesting that the outlier substantially influenced the P-TES value. These differences can be examined by comparing Table 1 with the S1 Table. For example, for the non-journal article sub-distribution of effect sizes, the P-TES value including the outlier was .53, but .95 with the outlier dropped. For the non-journal articles which used a non-contextualized measure, the P-TES was .65 when including the outlier and .76 without the outlier. When the purpose of the measure was classified as general purpose, P-TES was .32 with the outlier and .84 without it. When the research design was concurrent, the P-TES value including the outlier was .12 and thus approached a value (.10) in which one might draw an inference of a non-credible data set. However, the P-TES rose to .49 when the outlier was removed from the data set. Based on these results, when P-TES is used as a sensitivity analysis in a meta-analysis, we recommend that it be conducted with and without outliers to determine the robustness of the results. Using the typical criterion .10 or less [44], neither the full distribution nor the sub-distributions were judged to be non-credible sets of data. Concerning the results reported in Table 1, we found varying degrees of robustness in the meta-analytic mean (i.e., validity) estimates for conscientiousness. For the entire distribution (*k* = 113), the RE meta-analytic mean estimate (.16) was robust to the one sample removed analyses (e.g., the mean estimate did not change). However, the 90% prediction interval, which indicates the likely range of “true” effect sizes, is relatively wide (.03, .29). Furthermore, the RE meta-analytic mean estimate was not robust to all publication bias analyses. Specifically, the trim and fill estimate of .13 and the severe selection model estimate of .12 were noticeably smaller in magnitude than the RE estimate. Confirming these results, the PET-PEESE estimate was .13 (because PET was significant, the PEESE adjusted mean estimate was selected [45]). The PET test (.09, *p* < .001) supports the results from the trim and fill analysis by indicating that the effect size distribution is asymmetric; that small magnitude effect sizes are likely to be missing from the meta-analytic distribution.

**Fig 1:**
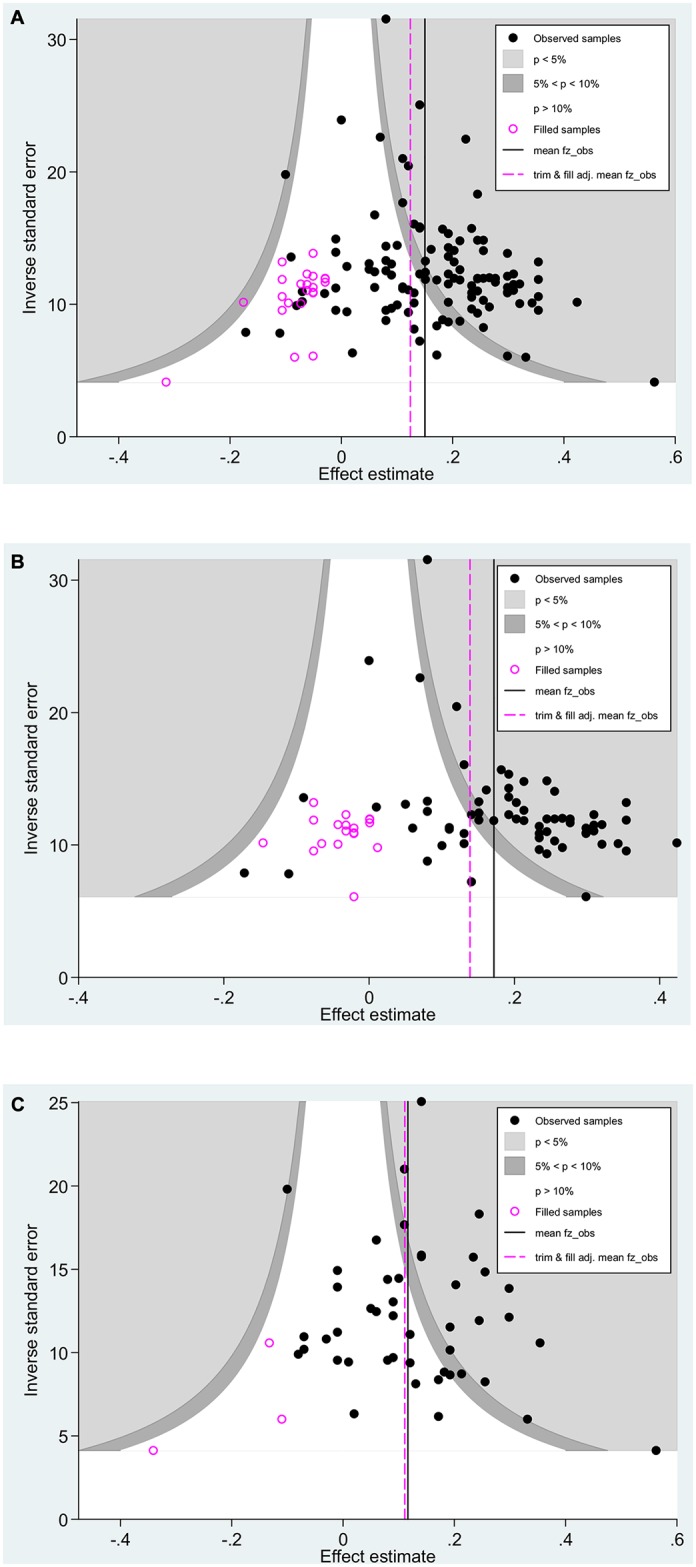
Three contoured funnel plots for the validity of conscientiousness by data source. (A) Conscientiousness data from all data sources. (B) Conscientiousness data from journal articles. (C) Conscientiousness data from non-journal sources. Correlations are graphed as circles with an X-axis of correlation magnitude and a Y-axis of the inverse standard error of the correlation. The filled black circles represent the observed correlations and the clear circles represent the trim-and-fill imputed correlations. The clear area contains correlations that are not statistically significant (*p* > .05). The darkest gray area contains correlations that may be described as marginally significant (*p*-values ranging from .05 to .10). The lighter gray area contains correlations that are statistically significant (*p* < .05). Note that most of the imputed correlations are found in the data distribution drawn from studies published in journals; relatively few of the imputed correlations are found in the data distribution drawn from unpublished studies. This fact is consistent with an inference that publication bias in the full data distribution is largely due to the suppression of statistically insignificant correlations in journal published articles. Thus, it is the journal articles that are largely responsible for distorting the research on the validity of conscientiousness.

**Fig 2:**
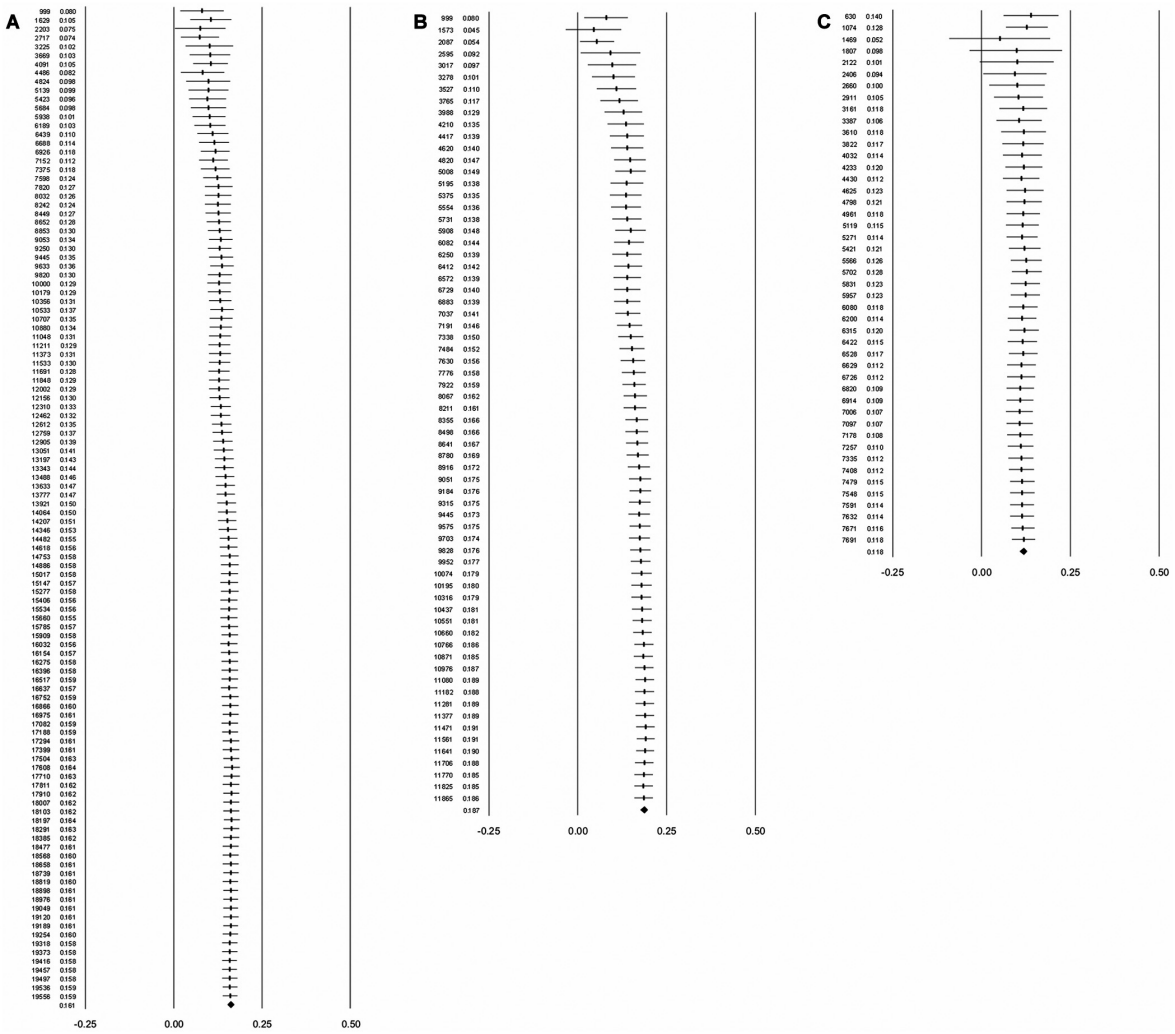
Three forest plots for the validity of conscientiousness by data source. (A) Conscientiousness data from all data sources. (B) Conscientiousness data from journal articles. (C) Conscientiousness data from non-journal sources. Forest plots for the cumulative meta-analyses by precision for the validity of conscientiousness (i.e., the correlation between conscientiousness and job performance) are displayed. To obtain the plots, validities were sorted from largest sample size to smallest sample size and entered into the meta-analysis one at a time in an iterative manner. The lines around the plotted means are the 95% confidence intervals for the meta-analytic means. For panels A and B, the mean validities drift from smaller to larger as correlations from smaller and smaller sample size studies are added the to the distribution being analyzed. For Panel C, no noticeable drift is observed. The drifts from smaller to larger meta-analytic means are consistent with an inference of statistically insignificant correlations from smaller sample size studies being suppressed (i.e., publication bias). The lack of meaningful drift in panel C suggests that the data suppression is largely in the journal published articles (see panel B). Thus, it is the data published in journal articles that are largely responsible for distorting the research on the validity of conscientiousness.

The contour-enhanced funnel plot (see Fig 1a) shows that all but one of the 23 imputed samples were in the area of statistical insignificance, which is consistent with an inference of publication bias stemming from the suppression of small magnitude correlations [33, 50]. The forest plot for the cumulative meta-analysis by precision shown in Fig 2a suggests that as sample sizes decrease, there is a noticeable drift toward higher validities. The cumulative point estimate starts at .07 (*N*_*cum*_ [cumulative sample size] = 2,717; *k*_*cum*_ [cumulative number of samples] = 4) with relatively large samples and increases to .13 (*N*_*cum*_ = 9,250; *k*_*cum*_ = 28) with the addition of smaller samples. Finally the validity estimate increases to .16 (*N*_*cum*_ = 19,625; *k*_*cum*_ = 113) with the addition of even smaller samples. This is consistent with an inference of publication bias resulting from the suppression of small magnitude correlations (from small samples). These patterns, especially the one from the contour-enhanced funnel plot, are also inconsistent with the notion that the small sample bias (i.e., small sample studies show systematic differences from larger sample studies due to assessing different populations or having measures of different sensitivity) is the cause for the observed results [33, 50]. We conclude that publication bias has likely affected the observed mean validity of conscientiousness for predicting job performance such that it is likely to be smaller in magnitude than the RE meta-analytic mean of .16. We note that most of the bias stems from journal articles (see Table 1 as well as Figs 1 and 2), which is consistent with an inference of the suppression of statistically non-significant results. Thus, it is the literature published in journals that is largely responsible for distorting the research on the validity of conscientiousness.

The results from the analyses without the outlier were similar. Therefore, we do not discuss the analyses or results without the one outlier. However, the results for all analyses without the outlier are provided in the supplementary materials (see S1 Table).

Our findings, including the range estimates (BRE and MRE) and conclusions, are summarized in Table 2 (S2 Table contains the conclusions for the sub-distributions without the sole outlier). We note that the range estimates are not necessarily perfectly comparable if the severe selection model did not provide a sensible solution (indicated by n/a in Table 1; see [41]). For these distributions, the observed range estimates may be smaller when compared to distributions where the full range of estimates is available. In addition, the results of the *p*-uniform analyses did not converge well with the results from the other, more established methods. Most likely, this is largely due to the heterogeneity in the data. The article that introduced *p*-uniform [47] provided simulation evidence that it noticeably overestimates the effect size as heterogeneity increases. We note that our *I* ^*2*^ values are typically near about 50, indicating non-trivial heterogeneity, which adversely affects the performance of *p*-uniform [47]. Correspondence with one of the authors of the article introducing the *p*-uniform method, while informative, did not result in a decision rule concerning the magnitude of *I* ^*2*^ values at which *p*-uniform should not be used [57]. Because of the nonconvergence with the results from the other, more established methods and our substantial uncertainty about the appropriateness of the *p*-uniform approach for these data (e.g., van Assen et al. [47] noted that this method performs poorly with heterogeneous data, which may explain why the *p*-uniform results generally did not converge with the other results), we excluded the results from our conclusions and Table 2 (and S4 Table; for conclusions of the results with the sole outlier that includes the results from the *p*-uniform analysis, see S3 Table).

**Table 2:** Robustness of results and conclusions of the analyses.

Distribution	Lowest value	${\overset{-}{r}}_{o_{RE}}$	Highest value	BRE	Practical difference	MRE	Practical difference	Conclusion ^a^
Conscientiousness	.12 ^f^	.16	.16 ^b^ ^,^ ^c^	.04 (25%)	moderate	.04 (25%)	moderate	Moderate difference
Frame of reference								
- Non-contextualized	.09 ^f^	.15	.16 ^c^	.06 (40%)	large	.07 (47%)	large	Large difference
- Contextualized	.17 ^d^ ^,^ ^g^	.19	.20 ^c^	.02 (11%)	negligible	.03 (16%)	negligible	Negligible difference
Source								
- Journal articles	.07 ^g^	.19	.19 ^b^ ^,^ ^c^	.12 (63%)	large	.12 (63%)	large	Large difference
- Non-contextualized	.07 ^g^	.19	.19 ^b^ ^,^ ^c^	.12 (63%)	large	.12 (63%)	large	Large difference
- Contextualized	.07 ^g^	.19	.20 ^c^	.12 (63%)	large	.13 (68%)	large	Large difference
- Non-journal articles	.10 ^e^	.12	.13 ^c^	.02 (17%)	negligible	.03 (25%)	moderate	Negligible to moderate difference
- Non-contextualized	.08 ^e^	.11	.11 ^b^ ^,^ ^c^ ^,^ ^d^ ^,^ ^g^	.03 (27%)	moderate	.03 (27%)	moderate	Moderate difference
- Contextualized	*Distribution is too small to reach definite conclusions regarding the robustness of the meta-analytic mean estimate*							
Purpose								
- General purpose	.08 ^f^	.14	.15 ^c^	.06 (43%)	large	.07 (50%)	large	Large difference
- Non-contextualized	.11 ^d^ ^,^ ^e^	.14	.15 ^c^	.03 (21%)	moderate	.04 (29%)	moderate	Moderate difference
- Contextualized	*Distribution is too small to reach definite conclusions regarding the robustness of the meta-analytic mean estimate*							
- Workplace purpose	.16 ^g^	.19	.20 ^c^	.03 (16%)	negligible	.04 (21%)	moderate	Negligible to moderate difference
- Non-contextualized	.09 ^g^	.19	.20 ^c^	.10 (53%)	large	.11 (58%)	large	Large difference
- Contextualized	.19 ^c^ ^,^ ^d^ ^,^ ^e^ ^,^ ^f^	.20	.21 ^c^	.01 (5%)	negligible	.02 (10%)	negligible	Negligible difference
Sample								
- Incumbents	.11 ^f^	.16	.16 ^b^ ^,^ ^c^	.05 (31%)	moderate	.05 (31%)	moderate	Moderate difference
- Non-contextualized	.09 ^f^	.15	.15 ^b^ ^,^ ^c^	.06 (40%)	large	.06 (40%)	large	Large difference
- Contextualized	.11 ^g^	.19	.20 ^c^	.08 (42%)	large	.09 (47%)	large	Large difference
- Applicants	*Distribution is too small to reach definite conclusions regarding the robustness of the meta-analytic mean estimate*							
- Non-contextualized	*Distribution is too small to reach definite conclusions regarding the robustness of the meta-analytic mean estimate*							
- Contextualized	*Distribution is too small to reach definite conclusions regarding the robustness of the meta-analytic mean estimate*							
Design								
- Concurrent design	.11 ^f^	.15	.16 ^c^	.04 (27%)	moderate	.05 (31%)	moderate	Moderate difference
- Non-contextualized	.09 ^f^	.15	.15 ^b^ ^,^ ^c^	.06 (40%)	large	.06 (40%)	large	Large difference
- Contextualized	.11 ^g^	.18	.19 ^c^	.07 (39%)	moderate	.08 (44%)	large	Moderate to large difference
- Predictive design	*Distribution is too small to reach definite conclusions regarding the robustness of the meta-analytic mean estimate*							
- Non-contextualized	*Distribution is too small to reach definite conclusions regarding the robustness of the meta-analytic mean estimate*							
- Contextualized	*Distribution is too small to reach definite conclusions regarding the robustness of the meta-analytic mean estimate*							
Scale								
- NEO	.08 ^f^ ^,^ ^g^	.14	.14 ^b^ ^,^ ^c^	.06 (43%)	large	.06 (43%)	large	Large difference
- PCI	.20 ^d^	.24	.25 ^c^	.04 (17%)	negligible	.05 (21%)	moderate	Negligible to moderate difference
- PSI	.21 ^e^ ^,^ ^f^	.22	.22 ^b^ ^,^ ^c^ ^,^ ^d^	.01 (5%)	negligible	.01 (5%)	negligible	Negligible difference

*Note*: Lowest value = lowest mean estimate from all analyses (${\overset{-}{r}}_{o_{RE}}$; osr, ${\overset{-}{r}}_{o_{FE}}$, t&f ${\overset{-}{r}}_{o}$, sm_m_ ${\overset{-}{r}}_{o}$, sm_s_ ${\overset{-}{r}}_{o}$, and PET-PEESE; we did not include the *p*-uniform values due to the lack of convergence with the results of the other, more established methods; likely due to the poor performance of this method with heterogeneous data [47]); ${\overset{-}{r}}_{o_{RE}}$ = random-effects weighted mean observed correlation (the potentially best mean estimate); Highest value = highest mean estimate from all analyses (${\overset{-}{r}}_{o_{RE}}$; osr, ${\overset{-}{r}}_{o_{FE}}$, t&f ${\overset{-}{r}}_{o}$, sm_m_ ${\overset{-}{r}}_{o}$, sm_s_ ${\overset{-}{r}}_{o}$, PET-PEESE); BRE = Baseline range estimate: the absolute range between ${\overset{-}{r}}_{o_{RE}}$ and the estimate farthest away (either the lowest or highest value); MRE = Maximum range estimate: the absolute range between the lowest or highest value. When calculating the relative difference of the range estimates, we used ${\overset{-}{r}}_{o_{RE}}$, the potentially best mean estimate, as the base (i.e., as 100%). Ideally, BRE and MRE should be identical. If not, outliers or other artifacts may have caused such differences. Practical difference: negligible = if the relative range (BRE or MRE) is smaller than 20%; moderate = if the relative range (BRE or MRE) is larger than 20%; large = if the relative range (BRE or MRE) is larger than 40% [33]. We note that, in a few instances, the range estimates are not necessarily comparable when the severe selection model did not provide a sensible solution (indicated by n/a in Table 1). For these distributions, the range estimates may be smaller in their magnitude when compared to distributions where the full range of estimates is available.

^a^ Conclusions of a negligible difference indicate that the meta-analytic mean estimate (i.e., ${\overset{-}{r}}_{o_{RE}}$) is likely to be robust. Conclusions of a moderate, moderate to large, or large difference indicates that the meta-analytic mean estimate (i.e., ${\overset{-}{r}}_{o_{RE}}$) is likely to be non-robust and could be misestimated (i.e., ${\overset{-}{r}}_{o_{RE}}$ could be under- or overestimated; typically overestimated in our analyses).

^b^ = value from ${\overset{-}{r}}_{o_{RE}}$;

^c^ = value from osr, ${\overset{-}{r}}_{o_{FE}}$;

^d^ = value from t&f ${\overset{-}{r}}_{o}$;

^e^ = value from sm_m_ ${\overset{-}{r}}_{o}$;

^f^ = value from sm_s_ ${\overset{-}{r}}_{o}$;

^g^ = value from PET-PEESE (value from PEESE if the PET value was significant, value from PET if it was not significant).

Based on the sum of evidence, we conclude that the conscientiousness data are not meaningfully influenced by a sole outlier. We also found that, in general, the data on conscientiousness are noticeably affected by publication bias. Thus, the apparent suppression of small magnitude effect sizes, which the contour-enhanced funnel plots indicated to lie predominantly in the area of statistical insignificance, likely has led to the overestimation of the validity of conscientiousness. The results for the sub-group distributions of samples from journal articles (*k* = 67) and non-journal sources (*k* = 46) support this notion because samples published in journal articles reported larger average effect size estimates (${\overset{-}{r}}_{o_{RE}}$ = .19) than samples from non-journal sources (${\overset{-}{r}}_{o_{RE}}$ = .12; see Table 1). Distributions involving journal articles tended to be the most non-robust as well, typically with differences of at least .10 and overestimations of more than 60% (see Table 2). For illustrative purposes, we also provide the contour-enhanced funnel plots for both of these distributions as well as the forest plots from the respective cumulative meta-analysis by precision (see Fig 1b and 1c as well asFig 2b and 2c). The contour-enhanced funnel plots and the cumulative meta-analyses by precision support an inference of publication bias and an overestimation of the mean validity for data from journal articles as well [33]. By contrast, the data from non-journal sources seems to be relatively robust to publication bias (see Table 2). Thus, it is the data from journal articles that are largely responsible for distorting the research on the validity of conscientiousness.

In addition, we found that the RE mean validity estimates for distributions involving contextualized measures of conscientiousness were sometimes more robust than the mean estimates for distributions involving non-contextualized measures. For the distribution of all non-contextualized measures of conscientiousness (*k* = 91), the 90% prediction interval ranged from .00 to .29. By contrast, the prediction interval for the distribution of contextualized measures (*k* = 22) ranged only from .16 to .22. However, for many other distributions, the contextualization of conscientiousness measures did not matter. Often contextualized and non-contextualized sub-distributions were non-robust to a similar degree (non-robust to a moderate or even large degree [see Table 2]).

Although one may argue that the absolute difference between the RE meta-analytic mean estimates and the publication bias adjusted mean estimates tend to be rather small in magnitude (i.e., approximately .06 for most distributions), the relative differences tend to be noticeable (i.e., typically greater than 30%) and may be interpreted as moderate in size [6, 33]. Furthermore, for data from journal articles, the overestimation appears to be large, for contextualized as well as non-contextualized measures of conscientiousness (see Tables 1 and 2).

Based on a reviewer request, statistical significance tests are provided in Table 3 for the moderator subgroups analyzed in Table 1. Results in S4 Table are for the data set with the sole outlier removed.

**Table 3:** Moderator statistical tests using the between-group *Q* test.

Distribution	Between-group *Q*	*p*-value
Frame of Reference: Non-contextualized vs. Contextualized	3.54	.06
Source: Journal articles vs. non-journal articles	9.73	.00
-Journal articles: Non-contextualized vs. Contextualized	0.00	1.00
-Non-Journal articles: Non-contextualized vs. Contextualized	7.65	.01
Purpose: General vs Workplace	6.88	.01
-Purpose: General: Non-contextualized vs. Contextualized	.89	.35
-Purpose: Workplace: Non-contextualized vs. Contextualized	.10	.75
Sample: Incumbents vs. Applicants	4.51	.03
-Sample: Incumbents: Non-contextualized vs. Contextualized	3.33	.07
-Sample: Applicants: Non-contextualized vs. Contextualized	.03	.86
Design: Concurrent vs. Predictive	8.50	.00
-Design: Concurrent: Non-contextualized vs. Contextualized	2.43	.12
-Design: Predictive: Non-contextualized vs. Contextualized	.08	.77
Scale: NEO vs. PCI vs. PSI	14.77	.00
-NEO vs. PCI	12.54	.00
-NEO vs. PSI	7.20	.01
-PCI vs. PSI	.42	.52

## Discussion

Publication bias and outliers can distort meta-analytic results and conclusions [3, 16, 27, 28, 33]. Unfortunately, most meta-analyses in the organizational sciences fail to conduct sensitivity analyses to assess the effect of these phenomena and do not report results regarding the robustness of meta-analytic findings [12, 33] even though the *American Psychological Association* [13, 14] and other scientific organizations (e.g., the Cochrane Collaboration [15]) recommend such analyses [36]. We note that even a journal published by the *American Psychological Association* (i.e., the *Journal of Applied Psychology*) seldom reports sensitivity analyses in their meta-analytic studies despite the recommendation of the organization that owns the journal. In this study, we used a variety of sensitivity analyses to assess the robustness of claims regarding the validity of conscientiousness for predicting job performance.

Overall, we conclude that the observed validity for conscientiousness is overestimated in the literature. This overestimation is primarily due to the influence of publication bias and not outliers. However, the lack of a distorting effect due to outliers may not be true for other literature areas and, in accordance with best meta-analytic practices [36], we encourage the use of outlier analyses in all meta-analytic reviews. We note that some sub-distributions were less robust than others (see Table 2). The non-contextualized sub-distribution (*k* = 91) misestimated the validity of conscientiousness to a large degree (40% to 47%; see Table 2). By contrast, effect sizes drawn from studies with contextualized conscientiousness measures (*k* = 22) seemed to be relatively robust and free of publication bias. However, such differences in the degree of robustness between non-contextualized and contextualized measures of conscientiousness were not always evident. Data from journals (*k* = 67) misestimated the validity of conscientiousness by a large degree (63%). Data from non-journal sources (*k* = 46) typically showed negligible to moderate degrees of publication bias, indicating that most of the apparent data suppression is associated with journal articles.

In addition to these findings, misestimation was evident with general purpose measures (*k* = 76) and was judged relatively large (43% to 50%). On the other hand, data drawn from studies that used a workplace purpose measure of conscientiousness were only negligibly or moderately affected by publication bias (*k* = 37; 16% to 21% misestimation), particularly if they involved contextualized measures (5% to 10%) as opposed to non-contextualized measures (53% to 58%). Misestimation was moderate for incumbent samples (*k* = 109; 31%). The degree of contextualization did not matter as both incumbents’ sub-distributions (incumbents and non-contextualized measures; incumbents and contextualized measures) were non-robust to a large degree (e.g., their meta-analytic means were misestimated by up to potentially over 40%; see Table 2).

The misestimation of concurrent designs (*k* = 105) was judged moderate (27% to 31%). As with the incumbent samples, the degree of contextualization did not matter as the meta-analytic means of both distributions were overestimated by up to a large degree (misestimation for concurrent designs and non-contextualized samples [*k* = 86]: .06 [40%]; misestimation for concurrent designs and contextualized samples [*k* = 19]: .07 [39%] to .08 [44%]). Finally, there were sufficient data for three specific measures. Validities based on the NEO Personality Inventory [58] showed large misestimation (43%) whereas there was negligible to moderate (17% to 21%) publication bias in validities drawn from the Personal Characteristics Inventory (PCI [59]) and negligible bias (5%) involving the Personal Style Inventory (PSI [60]).

Based on our findings, we conclude that the validity estimates from non-journal sources are likely to be more robust than estimates from journal articles. Although the goal of our paper was not a critique of Shaffer and Postlethwaite’s study [31], the presence of potentially severe publication bias in samples from journal articles indicates that Shaffer and Postlethwaite overestimated the validity of conscientiousness measures, especially for non-contextualized measures of conscientiousness but also for contextualized ones. With regard to particular measures, it appears that the mean validity estimate for the NEO was comparatively low (e.g., ${\overset{-}{r}}_{o_{RE}}\;$= .14 vs. .24 and .22 for PCI and PSI measures, respectively) and non-robust (e.g., the estimate from the severe selection model is .08, suggesting that the RE mean estimate for NEO measure, already lower in magnitude than the estimate from the PCI and PSI (.14, .24, and .22, respectively), was overestimated by 43%). By contrast, the PCI and PSI measures seem to have validity estimates that are relatively robust and larger in magnitude.

It is possible that the NEO shows more publication bias than other measures because the NEO is a commercial employment test product while at least some of the other measures in the analysis are not commercially sold. McDaniel, Rothstein, and Whetzel drew inferences consistent with a conclusion of publication bias when examining several commercially sold employment tests [61]. They speculated that results that may damage the marketing of commercial products might be suppressed. The validity distribution of “Test Vendor A” had evaluated potential publication bias in the PCI, and consistent with the current paper’s results, found no compelling evidence of publication bias.

### Recommendations and limitations

There are several possible critiques of this research, two of which are described below. First, we note that some might argue that differences expressed in percentages might be better expressed as differences in correlation magnitude. Our tables present both. Second, some might argue that a correlation inflation of some magnitude (e.g., .05) due to publication bias (and/or outliers) is a small difference and not likely to be meaningful. In the context of predicting job performance, one approach to assess the meaningfulness is to calculate the dollar value of differences in validity.

Using the mean validity estimate for data from journal articles (*r* = .19) compared to the trim and fill adjusted estimate (*r* = .14), we compute the dollar value on assuming one value over the other. For these calculations, we used 40% of salary as the estimate of the standard deviation of job performance in dollars following Hunter and Schmidt [62] and $44,888 as the average salary in the United States [63]. We estimated the standard deviation of job performance in dollars as .4 * 44,888 = $17,955.20. We assumed that the average test performance of those hired is the score at the 85^th^ percentile of those completing a conscientiousness measure. Using a common utility formula (formula 1 in [64]) and assuming 100 employees were hired who work for 20 years, the utility value for a validity of .19 is about $1,800,000 larger than the utility value of a validity of .14. Thus, the use of incorrectly inflated validity coefficients due to publication bias or other phenomena sharply overestimates the dollar utility of personnel selection by millions of dollars. This should be of considerable concern for organizations. We acknowledge that different assumed values yield different results. For example, in more recent cohorts of employees, one may observe that few employees stay in an organization or job for 20 years. Thus, our estimates could be modified by considering repeated costs per hire. Yet, any reasonable values will show sharp overestimates of the utility in dollars when validity estimates are overestimated due to publication bias. Furthermore, we note that more sophisticated utility analyses (e.g., analyses with multiple predictors) could be conducted. Our simple utility analysis is offered to show that effect size differences of around .05 are not necessarily trivial in magnitude.

We have drawn inferences about publication bias in part from small sample studies having relatively large magnitude effect sizes when compared to large sample studies, on average. The assertion of publication bias is also supported by the contour-enhanced funnel plot and the cumulative meta-analyses by precision (see Figs 1 and 2). Here, we consider the alternative explanation that the mean effect size differences between small and large sample studies is due to “true” differences between such studies and thus not due to publication bias. One example scenario concerning “true” differences in small vs. large studies is from the medical literature, in which small sample studies may be drawn from a different population than larger sample studies. In medical interventions, small samples might be drawn from a population of very ill patients and result in larger effects sizes than larger samples, which may be drawn from a population of less ill patients. However, we have no theoretical or empirical evidence that the samples for the conscientiousness-job performance relation are drawn from such different populations.

A second scenario for “true” differences in small and large samples concerns the sensitivity of the measures. Consider a study assessing stress effects on humans. In smaller studies, it may be financially feasible to collect physiological measures of relevance to stress. However, such measures are likely more costly than self-reports of the effects of stress. In large sample studies, self-report survey data may be more common because the potentially more sensitive physiological measures are financially infeasible to collect in large samples. This may result in larger effects for the smaller studies than for the larger studies. However, in our study, the self-report measures used for smaller and larger studies are not distinguishable. Based on this reasoning, the evidence from the contour-enhanced funnel plot, and the fact that our results indicate that virtually all of the sample suppression is evident in data drawn from journal articles as opposed to non-journal sources, we conclude that “true” differences between the smaller and larger studies, other than sample size, are unlikely to be credible. Thus, we are confident that the difference in mean effects between small and large studies is best attributed to publication bias.

We suggest that meta-analytic researchers present a range of parameter estimates rather than a single point estimate [38, 65, 66]. In the context of meta-analytic reviews, triangulation means the use of multiple meta-analytic estimates, outlier identification, and publication bias detection methods to estimate the range of results rather than relying on a single point estimate [33]. According to Orlitzky [38], the use of multiple estimates permits the triangulation of results, which is important in advancing the methodological rigor in the organizational sciences and in obtaining more accurate and trustworthy results [10]. This approach is aligned with customer-centric reporting as researchers and practitioners benefit from understanding the robustness of a meta-analytic estimate [36, 67]. This recommendation is also supported by the Meta-analysis Reporting Standards of the *American Psychological Association* [13, 14, 36] and other scientific organizations, such as the *Cochrane Collaboration* [15].

If space considerations in journals prohibit detailed reporting of results, they should be made available on journal websites as supplementary information, a practice that is common in the medical sciences [68] and cross-disciplinary journals such as PLoS ONE. We suggest that such practices should become more common in psychology and management journals. Robust and non-robust estimates are equally informative about meta-analytic results and the associated conclusions. In the former case, the findings provide assurance regarding the accuracy of the meta-analytic estimates. In the latter case, non-robust results aid in the re-evaluation and revision of previously made conclusions, thereby directing new research efforts.

With regard to specific methodological recommendations for the detection of outliers, we suggest the use of the one-sample removed analysis to empirically assess the influence of each individual sample on meta-analytic results [37]. This analysis provides a range of results. We also recommend outlier analyses and the reporting of results with and without outliers. We reported the results using Viechtbauer and Cheung’s approach for outlier detection [48] with the diagnostic measures and criteria for determining whether a study is an outlier described by Viechtbauer [49]. In addition, we used Beal and colleagues SAMD statistic to identify outliers [69], which yielded very similar results and essentially identical conclusions (we conducted the SAMD analyses at the sub-distribution level of analysis). However, due to potential problems with the SAMD approach when the data are heterogeneous (the approach does not take [residual] heterogeneity into account; we thank an anonymous reviewer for highlighting this issue), we did not report the results.

Regarding the publication bias assessment methods, it is important to note that funnel plot-based methods (e.g., contour-enhanced funnel plot and trim and fill) are based on the degree of asymmetry in the funnel plot. Publication bias is one possible cause for the observed distribution asymmetry. Outliers and heterogeneity, either due to moderators or “true” differences between small and large samples (i.e., the small sample bias; [33, 50]), are other possible causes. We accounted for the possible heterogeneous effects of outliers by running all analyses with and without the sole outlier. We used the contour-enhanced funnel plot to distinguish publication bias from other causes of funnel plot asymmetry [39, 50]. As noted previously, we used the originally identified moderators by Shaffer and Postlethwaite and formed sub-distributions to reduce the degree of between-sample heterogeneity [51, 70], minimizing the possibility that funnel plot asymmetry resulted from this type of heterogeneity [33, 70, 71]. Also, our results were relatively consistent: the distributions with data from journal articles displayed noticeable publication bias while the distributions with data from non-journal sources showed negligible bias. Furthermore, all distributions involving non-contextualized measures of conscientiousness were affected by publication bias; their meta-analytic mean estimates were always non-robust. Thus, it seems unlikely that heterogeneity caused our results.

In addition, results of selection models, which are less affected by heterogeneity [33, 41, 42], provided supporting results and should receive considerable weight when estimating the effect of publication bias on meta-analytic results [33]. For virtually all distributions, the varying publication bias methods yielded similar results. A key exception were the results from the *p*-uniform analyses. They did not converge well with the results of any of the other, more established methods. The *p*-uniform method may have been inappropriate for our data set given the degree of heterogeneity [47]. Given that the degree of heterogeneity tends to be similar in other areas in applied psychology and management, it may be that *p*-uniform is not appropriate for most data sets in these research areas. Future research should investigate this issue. Similar caveats may apply to the PET-PEESE analysis [53], although our results tended to converge relatively well, especially when compared to the *p*-uniform results. Finally, as discussed previously, we are not aware of any empirical evidence or theoretical rationale to suggest that the small sample bias has caused our results. This conclusion is also supported by the patterns of the contour-enhanced funnel plots [33, 50].

Our findings are aligned with previous warnings regarding the influence of phenomena such as publication bias and outliers in meta-analytic reviews [6, 15, 27, 28, 61, 72–75]. Given our results, we argue that suggestions regarding the irrelevance of sensitivity analyses, particularly publication bias analyses [76], are clearly incorrect for the conscientiousness literature. We thus advocate comprehensive sensitivity analyses in all meta-analytic reviews to determine the degree of potential misestimation in meta-analytic results [11, 13, 14, 36]. We note that outliers and/or publication bias may not be present in all meta-analytic reviews. For example, the sole outlier did not affect our results and publication bias did affect the data from journal articles noticeably more than data from non-journal sources. Even when outliers and/or publication bias are present, they may not substantially affect the results and conclusions of all meta-analytic distributions and results. However, these phenomena may have a substantial effect on some meta-analytic findings. Thus, we recommend that sensitivity assessments always be reported in journal articles or the articles’ supplementary materials, regardless of whether or not outliers and publication and related biases affect meta-analytic results.

Currently, we do not know the degree to which phenomena such as outliers and publication bias have affected our cumulative knowledge. To provide such information and more accurate meta-analytic results, we support calls for comprehensive sensitivity analyses in all meta-analytic reviews, which is aligned with recommendations from the Meta-analysis Reporting Standards of the *American Psychological Association* [13, 14] and previous research efforts [3, 11, 33, 36, 75].

Researchers in applied psychology and the organizational sciences typically know the extent of measurement error in their data and sometimes have information on range variation on variables of interest. With such information, researchers can use psychometric meta-analysis methods [35] to obtain mean estimates of effects that would be obtained in the absence of measurement error and range restriction (or range enhancement). Such estimates are useful for studying relations among variables at the construct or latent level, contribute to theory clarifications, and are valuable in practical applications (e.g., comparisons of the value of various employment screening procedures). Unfortunately, current publication bias methods have not been designed with psychometrically-adjusted effect sizes in mind. Nor are the publication bias methods accommodating to psychometric meta-analytic perspectives on study weighting (sample size vs. inverse variance weighting), effect size transformations (i.e., Fisher *z*), and sampling error estimation (i.e., estimate of rho in sampling error estimates). Psychometric meta-analysis methods that correct individual effect sizes for measurement error and range variation issues yield effect sizes that could be used in current publication bias methods if the standard errors are appropriately adjusted (and the methods do not estimate sampling error from sample size and the observed effect size). Still, there is no current research assessing the accuracy of publication bias methods using the psychometric approach. Also, to our knowledge, there are no publication bias methods that are adaptable to psychometric meta-analysis approaches using artifact distributions. We encourage efforts to evaluate current methods of publication bias for psychometric meta-analysis applications.

## Conclusions

Sensitivity analyses are rarely performed in the organizational sciences [12, 29, 72]. Despite suggestions to the contrary [76], we found that publication bias can have noticeable effects on meta-analytic results. Our findings illustrate the need for a rigorous quantitative assessment of the robustness of meta-analytic results. Errors in primary studies are problematic, but those affecting the conclusions of a meta-analytic review can mislead future research directions and misinform evidence-based practice [3, 4, 33]. We encourage the use of methods for the detection of outliers and publication bias in all meta-analytic reviews, and, aligned with the approach of triangulation [38, 65, 66] and customer-centric science [67], the reporting of the range of results. Journals, which should provide the best estimates of effect sizes, are, ironically, providing the most biased estimates. Clearly, journal polices and the behavior of authors responding to journal policies, are in need of substantial revision [10].

## Supporting Information

S1 TableMeta-analytic and publication bias results (outlier excluded).
*k* = number of correlation coefficients in the analyzed distribution. Publication bias analyses were not conducted for distributions with less than *k* = 10; ${\overset{-}{r}}_{o_{RE}}$ = random-effects weighted mean observed correlation; 95% CI = 95% confidence interval; 90% PI = 90% prediction interval; *Q* = weighted sum of squared deviations from the mean; *I* ^2^ = ratio of true heterogeneity to total variation; *τ* = between-sample standard deviation; osr = one-sample removed, including the minimum and maximum effect size and the median weighted mean observed correlation; Trim and fill = trim and fill analysis; FPS = funnel plot side (i.e., side of the funnel plot where samples were imputed; L = left, R = right); *ik* = number of trim and fill imputed samples; t&f ${\overset{-}{r}}_{o}$ = trim and fill adjusted observed mean (the weighted mean of the distribution of the combined observed and the imputed samples); t&f 95% CI = trim and fill adjusted 95% confidence interval; sm_m_ ${\overset{-}{r}}_{o}$ = one-tailed moderate selection model’s adjusted observed mean (and its variance); sm_s_ ${\overset{-}{r}}_{o}$ = one-tailed severe selection model’s adjusted observed mean (and its variance); Ex. sig. = excess significance; PET-PEESE = precision-effect test-precision effect estimate with standard error; PET = PET adjusted observed mean (and its one-tailed *p*-value; the value from PEESE is the adjusted observed mean if the PET value is significant, the value from PET is the adjusted observed mean if the *p*-value is not significant [45]); PEESE = PEESE adjusted observed mean; P-TES = the probability of the chi-square test of excess significance; *p*-uniform (95% CI) = the *p*-uniform estimate and its 95% confidence interval; n/a = not applicable (because *k* was too small to conduct these analyses or because the variance component for the selection models indicated that the estimate was nonsensical [33]).(DOCX)

S2 TableRobustness of results and conclusions of the analyses (outlier excluded).Lowest value = lowest mean estimate from all analyses (${\overset{-}{r}}_{o_{RE}}$; osr, ${\overset{-}{r}}_{o_{FE}}$, t&f ${\overset{-}{r}}_{o}$, sm_m_ ${\overset{-}{r}}_{o}$, sm_s_ ${\overset{-}{r}}_{o}$, and PET-PEESE; we did not include the *p*-uniform values due to the lack of convergence with the results of the other, more established methods; likely due to the poor performance of this method with heterogeneous data [van Assen et al., in press]); ${\overset{-}{r}}_{o_{RE}}$ = random-effects weighted mean observed correlation (the potentially best mean estimate); Highest value = highest mean estimate from all analyses (${\overset{-}{r}}_{o_{RE}}$; osr, ${\overset{-}{r}}_{o_{FE}}$, t&f ${\overset{-}{r}}_{o}$, sm_m_ ${\overset{-}{r}}_{o}$, sm_s_ ${\overset{-}{r}}_{o}$, PET-PEESE); BRE = Baseline range estimate: the absolute range between ${\overset{-}{r}}_{o_{RE}}$ and the estimate farthest away (either the lowest or highest value); MRE = Maximum range estimate: the absolute range between the lowest or highest value. When calculating the relative difference of the range estimates, we used ${\overset{-}{r}}_{o_{RE}}$, the potentially best mean estimate, as the base (i.e., as 100%). Ideally, BRE and MRE should be identical. If not, outliers or other artifacts may have caused such differences. Practical difference: negligible = if the relative range (BRE or MRE) is smaller than 20%; moderate = if the relative range (BRE or MRE) is larger than 20%; large = if the relative range (BRE or MRE) is larger than 40% [33].(DOCX)

S3 TableRobustness of results and conclusions of the analyses (including *p*-uniform estimates).Lowest value = lowest mean estimate from all analyses (${\overset{-}{r}}_{o_{RE}}$; osr, ${\overset{-}{r}}_{o_{FE}}$, t&f ${\overset{-}{r}}_{o}$, sm_m_ ${\overset{-}{r}}_{o}$, sm_s_ ${\overset{-}{r}}_{o}$, PET-PEESE, and *p*-uniform); ${\overset{-}{r}}_{o_{RE}}$ = random-effects weighted mean observed correlation (the potentially best mean estimate); Highest value = highest mean estimate from all analyses (${\overset{-}{r}}_{o_{RE}}$; osr, ${\overset{-}{r}}_{o_{FE}}$, t&f ${\overset{-}{r}}_{o}$, sm_m_ ${\overset{-}{r}}_{o}$, sm_s_ ${\overset{-}{r}}_{o}$, PET-PEESE, and *p*-uniform); BRE = Baseline range estimate: the absolute range between ${\overset{-}{r}}_{o_{RE}}$ and the estimate farthest away (either the lowest or highest value); MRE = Maximum range estimate: the absolute range between the lowest or highest value. When calculating the relative difference of the range estimates, we used ${\overset{-}{r}}_{o_{RE}}$, the potentially best mean estimate, as the base (i.e., as 100%). Ideally, BRE and MRE should be identical. If not, outliers or other artifacts may have caused such differences. Practical difference: negligible = if the relative range (BRE or MRE) is smaller than 20%; moderate = if the relative range (BRE or MRE) is larger than 20%; large = if the relative range (BRE or MRE) is larger than 40% [Kepes et al., 2012].(DOCX)

S4 Table Moderator statistical tests using the between group *Q* test (outlier excluded). (DOCX)

## References

[1] Briner RB, Rousseau DM. Evidence-based I-O psychology: Not there yet. Industrial and Organizational Psychology: Perspectives on Science and Practice. 2011;4(1):3–22. 10.1111/j.1754-9434.2010.01287.x

[2] Le H, Oh I-S, Shaffer J, Schmidt FL. Implications of methodological advances for the practice of personnel selection: How practitioners benefit from meta-analysis. Academy of Management Perspectives. 2007;21(3):6–15.

[3] Banks GC, McDaniel MA. The kryptonite of evidence-based I-O psychology. Industrial and Organizational Psychology: Perspectives on Science and Practice. 2011;4(1):40–4. 10.1111/j.1754-9434.2010.01292.x

[4] Kepes S, Bennett AA, McDaniel MA. Evidence-based management and the trustworthiness of our cumulative scientific knowledge: Implications for teaching, research, and practice. Academy of Management Learning & Education. 2014;13(3):446–66.

[5] Fiedler K. Voodoo correlations are everywhere—Not only in neuroscience. Perspectives on Psychological Science. 2011;6(2):163–71. 10.1177/1745691611400237 26162135

[6] Rothstein HR, Sutton AJ, Borenstein M. Publication bias in meta-analyses In: Rothstein HR, Sutton AJ, Borenstein M, editors. Publication bias in meta-analysis: Prevention, assessment, and adjustments. West Sussex, UK: Wiley; 2005 p. 1–7.

[7] Viswesvaran C, Barrick MR, Ones DS. How definitive are conclusions based on survey data: Estimating robustness to nonresponse. Personnel Psychology. 1993;46(3):551–67. 10.1111/j.1744-6570.1993.tb00884.x

[8] Gilboa S, Shirom A, Fried Y, Cooper C. A meta-analysis of work demand stressors and job performance: Examining main and moderating effects. Personnel Psychology. 2008;61(2):227–71. 10.1111/j.1744-6570.2008.00113.x

[9] Hartshorne J, Schachner A. Tracking replicability as a method of post-publication open evaluation. Frontiers in Computational Neuroscience. 2012;6:1–14. 10.3389/fncom.2012.00008 22403538 PMC3293145

[10] Kepes S, McDaniel MA. How trustworthy is the scientific literature in industrial and organizational psychology? Industrial and Organizational Psychology: Perspectives on Science and Practice. 2013;6(3):252–68. 10.1111/iops.12045

[11] Greenhouse JB, Iyengar S. Sensitivity analysis and diagnostics In: Cooper H, Hedges LV, Valentine JC, editors. The handbook of research synthesis and meta-analysis. 2nd ed New York, NY: Russell Sage Foundation; 2009 p. 417–33.

[12] Aytug ZG, Rothstein HR, Zhou W, Kern MC. Revealed or concealed? Transparency of procedures, decisions, and judgment calls in meta-analyses. Organizational Research Methods. 2012;15(1):103–33. 10.1177/1094428111403495

[13] American Psychological Association. Reporting standards for research in psychology: Why do we need them? What might they be? American Psychologist. 2008;63(9):839–51. 10.1037/0003-066X.63.9.839 19086746 PMC2957094

[14] American Psychological Association. Publication manual of the American Psychological Association. 6th ed Washington, DC: American Psychological Association; 2010.

[15] Higgins JP, Green S, editors. Cochrane handbook for systematic reviews of interventions; Version 5.1.0 [updated March 2011]: The Cochrane Collaboration. Available from www.cochrane-handbook.org; 2011.

[16] Banks GC, Kepes S, McDaniel MA. Publication bias: Understanding the myths concerning threats to the advancement of science In: Lance CE, Vandenberg RJ, editors. More statistical and methodological myths and urban legends. New York, NY: Routledge; 2015 p. 36–64.

[17] van Lent M, Overbeke J, Out HJ. Role of editorial and peer review processes in publication bias: Analysis of drug trials submitted to eight medical journals. PLoS ONE. 2014;9(8):e104846 10.1371/journal.pone.0104846 25118182 PMC4130599

[18] Kicinski M. Publication bias in recent meta-analyses. PLoS ONE. 2013;8(11):e81823 10.1371/journal.pone.0081823 24363797 PMC3868709

[19] Sena ES, van der Worp HB, Bath PMW, Howells DW, Macleod MR. Publication bias in reports of animal stroke studies leads to major overstatement of efficacy. PLoS Biology. 2010;8(3):e1000344 10.1371/journal.pbio.1000344 20361022 PMC2846857

[20] ter Riet G, Korevaar DA, Leenaars M, Sterk PJ, Van Noorden CJF, Bouter LM, et al Publication bias in laboratory animal research: A survey on magnitude, drivers, consequence and potential solutions. PLoS ONE. 2012;7(9):e43404 10.1371/journal.pone.0043404 22957028 PMC3434185

[21] Dickersin K. The existence of publication bias and risk factors for Its occurrence. Journal of the American Medical Association. 1990;263(10):1385–9. 2406472

[22] Dickersin K. Publication bias: Recognizing the problem, understandings its origins and scope, and preventing harm In: Rothstein HR, Sutton AJ, Borenstein M, editors. Publication bias in meta-analysis: Prevention, assessment, and adjustments. West Sussex, UK: Wiley; 2005 p. 11–34.

[23] Sutton AJ. Evidence concerning the consequences of publication and related biases In: Rothstein HR, Sutton AJ, Borenstein M, editors. Publication bias in meta-analysis: Prevention, assessment, and adjustments. West Sussex, UK: Wiley; 2005 p. 175–92.

[24] Franco A, Malhotra N, Simonovits G. Publication bias in the social sciences: Unlocking the file drawer. Science. 2014;345(6203):1502–5. 10.1126/science.1255484 25170047

[25] Chalmers I, Dickersin K. Biased under-reporting of research reflects biased under-submission more than biased editorial rejection. F1000Research. 2013;2:1–6. 10.3410/f1000research.2-1.v1 24358860 PMC3782352

[26] Nosek BA, Spies JR, Motyl M. Scientific utopia: II. Restructuring incentives and practices to promote truth over publishability. Perspectives on Psychological Science. 2012;7(6):615–31. 10.1177/1745691612459058 26168121 PMC10540222

[27] Huffcutt AI, Arthur W. Development of a new outlier statistic for meta-analytic data. Journal of Applied Psychology. 1995;80:327–34. 310.1037/0021-9010.1080.1032.1327.

[28] Schmidt FL, Law K, Hunter JE, Rothstein HR, Pearlman K, McDaniel M. Refinements in validity generalization methods: Implications for the situational specificity hypothesis. Journal of Applied Psychology. 1993;78(1):3–12. 10.1037/0021-9010.78.1.3

[29] Aguinis H, Pierce CA, Bosco FA, Dalton DR, Dalton CM. Debunking myths and urban legends about meta-analysis. Organizational Research Methods. 2011;14(2):306–31. 10.1177/1094428110375720

[30] Digman JM. Personality structure: Emergence of the five-factor model. Annual Review of Psychology. 1990;41:417–40. 10.1146/annurev.ps.41.020190.002221

[31] Shaffer JA, Postlethwaite BE. A matter of context: A meta-analytic investigation of the relative validity of contextualized and noncontextualized personality measures. Personnel Psychology. 2012;65(3):445–93. 10.1111/j.1744-6570.2012.01250.x

[32] Borenstein M, Hedges LV, Higgins JP, Rothstein HR. Comprehensive meta-analysis (Version 2). Englewood, NJ: Biostat; 2005.

[33] Kepes S, Banks GC, McDaniel MA, Whetzel DL. Publication bias in the organizational sciences. Organizational Research Methods. 2012;15(4):624–62. 10.1177/1094428112452760

[34] Hedges LV, Olkin I. Statistical methods for meta-analysis. New York, NY: Academic Press; 1985.

[35] Schmidt FL, Hunter JE. Methods of meta-analysis: Correcting error and bias in research findings. 3rd ed Newbury Park, CA: Sage; 2015.

[36] Kepes S, McDaniel MA, Brannick MT, Banks GC. Meta-analytic reviews in the organizational sciences: Two meta-analytic schools on the way to MARS (the Meta-Analytic Reporting Standards). Journal of Business and Psychology. 2013;28(2):123–43. 10.1007/s10869-013-9300-2

[37] Borenstein M, Hedges LV, Higgins JP, Rothstein HR. Introduction to meta-analysis. West Sussex, UK: Wiley; 2009.

[38] Orlitzky M. How can significance tests be deinstitutionalized? Organizational Research Methods. 2012;15(2):199–228. 10.1177/1094428111428356

[39] Peters JL, Sutton AJ, Jones DR, Abrams KR, Rushton L. Contour-enhanced meta-analysis funnel plots help distinguish publication bias from other causes of asymmetry. Journal of Clinical Epidemiology. 2008;61(10):991–6. 10.1016/j.jclinepi.2007.11.010 18538991

[40] Duval SJ. The “trim and fill” method In: Rothstein HR, Sutton AJ, Borenstein M, editors. Publication bias in meta-analysis: Prevention, assessment, and adjustments. West Sussex, UK: Wiley; 2005 p. 127–44.

[41] Vevea JL, Woods CM. Publication bias in research synthesis: Sensitivity analysis using a priori weight functions. Psychological Methods. 2005;10:428–43. 10.1037/1082-989X.10.4.428 16392998

[42] Field AP, Gillett R. How to do a meta-analysis. British Journal of Mathematical and Statistical Psychology. 2010;63:665–94. 10.1348/000711010X502733 20497626

[43] Ioannidis JP, Trikalinos TA. An exploratory test for an excess of significant findings. Clinical Trials. 2007;4(3):245–53. 10.1177/1740774507079441 17715249

[44] Francis G. The frequency of excess success for articles in Psychological Science. Psychonomic Bulletin & Review. 2014;21(5):1180–7. 10.3758/s13423-014-0601-x 24638826

[45] Stanley TD, Doucouliagos H. Meta-regression approximations to reduce publication selection bias. Research Synthesis Methods. 2014;5(1):60–78. 10.1002/jrsm.1095 26054026

[46] Egger M, Smith GD, Schneider M, Minder C. Bias in meta-analysis detected by a simple, graphical test. British Medical Journal. 1997;315(7109):629–34. 10.1136/bmj.315.7109.629 9310563 PMC2127453

[47] van Assen MALM, van Aert RCM, Wicherts JM. Meta-analysis using effect size distributions of only statistically significant studies. Psychological Methods. 2015;20(3):293–309. 10.1037/met0000025 25401773

[48] Viechtbauer W, Cheung MWL. Outlier and influence diagnostics for meta-analysis. Research Synthesis Methods. 2010;1(2):112–25. 10.1002/jrsm.11 26061377

[49] Viechtbauer W. Meta-analysis package for R: Package ‘metafor.’ R package version 1.9–5. 2015.

[50] Sterne JAC, Sutton AJ, Ioannidis JPA, Terrin N, Jones DR, Lau J, et al Recommendations for examining and interpreting funnel plot asymmetry in meta-analyses of randomised controlled trials. British Medical Journal. 2011;343:d4002 10.1136/bmj.d4002 21784880

[51] Terrin N, Schmid CH, Lau J, Olkin I. Adjusting for publication bias in the presence of heterogeneity. Statistics in Medicine. 2003;22(13):2113–26. 10.1002/sim.1461 12820277

[52] Peters JL, Sutton AJ, Jones DR, Abrams KR, Rushton L. Performance of the trim and fill method in the presence of publication bias and between-study heterogeneity. Statistics in Medicine. 2007;26(25):4544–62. 10.1002/sim.2889 17476644

[53] Moreno SG, Sutton AJ, Ades AE, Stanley TD, Abrams KR, Peters JL, et al Assessment of regression-based methods to adjust for publication bias through a comprehensive simulation study. BMC Medical Research Methodology. 2009;9:2 10.1186/1471-2288-9-2 19138428 PMC2649158

[54] O'Boyle EH, Rutherford MW, Banks GC. Publication bias in entrepreneurship research: An examination of dominant relations to performance. Journal of Business Venturing. 2014;29(6):773–84. 10.1016/j.jbusvent.2013.10.001

[55] Lao RY-R. Faking in personality measures: Effects on the prediction of job performance [Unpublished doctoral dissertation]: North Carolina State University; 2001.

[56] Hirsh HR, Northrop LC, Schmidt FL. Validity generalization results for law enforcement occupations. Personnel Psychology. 1986;39(2):399–420. 10.1111/j.1744-6570.1986.tb00589.x

[57] van Aert RCM. Personal communication to to M. A. McDaniel. 2015.

[58] Costa PT, McCrae RR. Revised NEO Personality Inventory (NEO PI-R) and NEO Five-Factor Inventory (NEO-FFI) professional manual. Odessa, FL: Psychological Assessment Resources; 1992.

[59] Mount MK, Barrick MR. Personal Characteristics Inventory. Libertyville, IL: Wonderlic, Inc; 2007.

[60] Lounsbury JW, Gibson LW. Personal Style Inventory: A personality measurement system for work and school settings. Knoxville, TN: Resource Associates, Inc; 2006.

[61] McDaniel MA, Rothstein HR, Whetzel DL. Publication bias: A case study of four test vendors. Personnel Psychology. 2006;59(4):927–53. 10.1111/j.1744-6570.2006.00059.x

[62] Hunter JE, Schmidt FL. Quantifying the effects of psychological interventions on employee job performance and work-force productivity. American Psychologist. 1983;38(4):473–8. 10.1037/0003-066X.38.4.473

[63] Social Security Administration. National average wage index. United States Social Security Administration official web site (http://wwwssagov/oact/cola/AWIhtml; accessed on January 14, 2015). 2013.

[64] Schmidt FL, Hunter JE, McKenzie RC, Muldrow TW. Impact of valid selection procedures on work-force productivity. Journal of Applied Psychology. 1979;64(6):609–26. 10.1037/0021-9010.64.6.609

[65] Jick TD. Mixing qualitative and quantitative methods: Triangulation in action. Administrative Science Quarterly. 1979;24:602–11. 10.2307/2392366

[66] Scandura TA, Williams EA. Research methodology in management: Current practices, trends, and implications for future research. The Academy of Management Journal. 2000;43(6):1248–64. 10.2307/1556348

[67] Aguinis H, Werner S, Abbott JL, Angert C, Park JH, Kohlhausen D. Customer-centric science: Reporting significant research results with rigor, relevance, and practical impact in mind. Organizational Research Methods. 2010;13(3):515–39. 10.1177/1094428109333339

[68] Evangelou E, Trikalinos TA, Ioannidis JP. Unavailability of online supplementary scientific information from articles published in major journals. The FASEB Journal. 2005;19:1943–4. 10.1096/fj.05-4784lsf 16319137

[69] Beal DJ, Corey DM, Dunlap WP. On the bias of Huffcutt and Arthur's (1995) procedure for identifying outliers in the meta-analysis of correlations. Journal of Applied Psychology. 2002;87(3):583–9. 10.1037/0021-9010.87.3.583 12090616

[70] Peters J, Sutton A, Jones DR, Abrams KR, Rushton L, Moreno SG. Assessing publication bias in meta-analyses in the presence of between-study heterogeneity. Journal of the Royal Statistical Society. 2010;173:575–91. 10.1111/j.1467-985X.2009.00629.x

[71] Sterne JA, Gavaghan D, Egger M. The funnel plot. In: Rothstein HR, Sutton AJ, Borenstein M, editors. Publication bias in meta-analysis: Prevention, assessment, and adjustments. West Sussex, UK: Wiley; 2005 p. 75–98.

[72] Banks GC, Kepes S, McDaniel MA. Publication bias: A call for improved meta-analytic practice in the organizational sciences. International Journal of Selection and Assessment. 2012;20(2):182–96. 10.1111/j.1468-2389.2012.00591.x

[73] Geyskens I, Krishnan R, Steenkamp J-BEM, Cunha PV. A review and evaluation of meta-analysis practices in management research. Journal of Management. 2009;35(2):393–419. 10.1177/0149206308328501

[74] Song F, Parekh S, Hooper L, Loke Y, Ryder J, Sutton AJ, et al Dissemination and publication of research findings: An updated review of related biases. Health Technology Assessment. 2010;14(8):1–220. 10.3310/hta14080 20181324

[75] Kepes S, Banks GC, Oh I-S. Avoiding bias in publication bias research: The value of "null" findings. Journal of Business and Psychology. 2014;29(2):183–203. 10.1007/s10869-012-9279-0

[76] Dalton DR, Aguinis H, Dalton CM, Bosco FA, Pierce CA. Revisiting the file drawer problem in meta-analysis: An assessment of published and non-published correlation matrices. Personnel Psychology. 2012;65(2):221–49. 10.1111/j.1744-6570.2012.01243.x

