# Supplementary material for: The Validity of Conscientiousness Is Overestimated in the Prediction of Job Performance
Source: PLoS One. 2015 Oct 30;10(10):e0141468. doi: 10.1371/journal.pone.0141468 (PMC4627756; doi:10.1371/journal.pone.0141468)
Supplement: S1 Table — k = number of correlation coefficients in the analyzed distribution. Publication bias analyses were not conducted for distributions with less than k = 10; r-oRE = random-effects weighted mean observed correlation; 95% CI = 95% confidence interval; 90% PI = 90% prediction interval; Q = weighted sum of squared deviations from the mean; I 2 = ratio of true heterogeneity to total variation; τ = between-sample standard deviation; osr = one-sample removed, including the minimum and maximum effect size and the median weighted mean observed correlation; Trim and fill = trim and fill analysis; FPS = funnel plot side (i.e., side of the funnel plot where samples were imputed; L = left, R = right); ik = number of trim and fill imputed samples; t&f r-o = trim and fill adjusted observed mean (the weighted mean of the distribution of the combined observed and the imputed samples); t&f 95% CI = trim and fill adjusted 95% confidence interval; smm r-o = one-tailed moderate selection model’s adjusted observed mean (and its variance); sms r-o = one-tailed severe selection model’s adjusted observed mean (and its variance); Ex. sig. = excess significance; PET-PEESE = precision-effect test-precision effect estimate with standard error; PET = PET adjusted observed mean (and its one-tailed p-value; the value from PEESE is the adjusted observed mean if the PET value is significant, the value from PET is the adjusted observed mean if the p-value is not significant [45]); PEESE = PEESE adjusted observed mean; P-TES = the probability of the chi-square test of excess significance; p-uniform (95% CI) = the p-uniform estimate and its 95% confidence interval; n/a = not applicable (because k was too small to conduct these analyses or because the variance component for the selection models indicated that the estimate was nonsensical [33]). (DOCX) [file pone.0141468.s001.docx]

**S1 Table. Meta-analytic and publication bias results (outlier excluded)**

|  | Meta-analysis | | | | | | | | Publication bias analyses | | | | | | | | | |
| --- | --- | --- | --- | --- | --- | --- | --- | --- | --- | --- | --- | --- | --- | --- | --- | --- | --- | --- |
|  |  |  | | | |  | | | Trim and fill | | | | Selection models | | Ex. sig. | PET-PEESE | | *p*-uniform |
| Distribution | *k* | $\bar{r}_{o_{RE}}$ | 95% CI | 90% PI | *Q* | *I*^2^ | *τ* | osr | FPS | *ik* | t&f $\bar{r}_{o}$ | t&f 95% CI | sm_m_ $\bar{r}_{o}$ | sm_s_ $\bar{r}_{o}$ | P-TES | PET | PEESE | (95% CI) |
| Conscientiousness | 112 | .16 | .14, .18 | .04, .28 | 211.42 | 47.50 | .074 | .16, .16; .16 | L | 17 | .14 | .12, .16 | .15 (.01) | .12 (.01) | .25 | .11 (.00) | .14 | .19 (.16,.22) |
| Frame of reference |  |  |  |  |  |  |  |  |  |  |  |  |  |  |  |  |  |  |
| - Non-contextualized | 90 | .15 | .13, .18 | .02, .28 | 186.66 | 52.32 | .080 | .15, .16; .15 | L | 13 | .13 | .10, .15 | .14 (.01) | .11 (.01) | .31 | .11 (.00) | .14 | .20 (.17,.23) |
| - Contextualized | *No outlier identified (see Table 1 for results)* | | | | | | | |  |  |  |  |  |  |  |  |  |  |
| Source |  |  |  |  |  |  |  |  |  |  |  |  |  |  |  |  |  |  |
| - Journal articles | *No outlier identified (see Table 1 for results)* | | | | | | | |  |  |  |  |  |  |  |  |  |  |
| - Non-contextualized | *No outlier identified (see Table 1 for results)* | | | | | | | |  |  |  |  |  |  |  |  |  |  |
| - Contextualized | *No outlier identified (see Table 1 for results)* | | | | | | | |  |  |  |  |  |  |  |  |  |  |
| - Non-journal articles | 45 | .13 | .10, .16 | .02, .23 | 72.22 | 39.08 | .064 | .12, .13; .13 |  | 0 | .13 | .10, .16 | .11 (.00) | .07 (.01) | .95 | .15 (.00) | .13 | .18 (.13,.23) |
| - Non-contextualized | 38 | .11 | .08, .15 | .00, .22 | 63.73 | 41.94 | .006 | .11, .12; .11 | R | 4 | .13 | .10, .16 | .10 (.00) | .04 (.01) | .76 | .17 (.00) | .13 | .19 (.13,.25) |
| - Contextualized | *No outlier identified (see Table 1 for results)* | | | | | | | |  |  |  |  |  |  |  |  |  |  |
| Purpose |  |  |  |  |  |  |  |  |  |  |  |  |  |  |  |  |  |  |
| - General purpose | 75 | .15 | .12, .17 | .01, .27 | 154.23 | 52.02 | .080 | .14, .15; .15 | L | 9 | .12 | .09, .15 | .13 (.01) | .09 (.01) | .84 | .11 (.01) | .13 | .20 (.16,.24) |
| - Non-contextualized | 68 | .14 | .11, .17 | .00, .28 | 148.96 | 55.02 | .085 | .14, .15; .14 | L | 6 | .12 | .09, .15 | .12 (.01) | .08 (.01) | .66 | .11 (.01) | .13 | .21 (.17,.25) |
| - Contextualized | *No outlier identified (see Table 1 for results)* | | | | | | | |  |  |  |  |  |  |  |  |  |  |
| - Workplace purpose | *No outlier identified (see Table 1 for results)* | | | | | | | |  |  |  |  |  |  |  |  |  |  |
| - Non-contextualized | *No outlier identified (see Table 1 for results)* | | | | | | | |  |  |  |  |  |  |  |  |  |  |
| - Contextualized | *No outlier identified (see Table 1 for results)* | | | | | | | |  |  |  |  |  |  |  |  |  |  |
| Sample |  |  |  |  |  |  |  |  |  |  |  |  |  |  |  |  |  |  |
| - Incumbents | 108 | .16 | .14, .18 | .04, .28 | 205.60 | 47.50 | .074 | .16, .16; .16 | L | 17 | .13 | .11, .16 | .14 (.00) | .12 (.01) | .41 | .11 (.00) | .14 | .19 (.16,.22) |
| - Non-contextualized | 87 | .15 | .13, .18 | .02, .28 | 181.76 | 52.69 | .081 | .15, .15; .15 | L | 11 | .13 | .10, .16 | .13 (.01) | .10 (.01) | .50 | .11 (.00) | .14 | .20 (.17,.24) |
| - Contextualized | *No outlier identified (see Table 1 for results)* | | | | | | | |  |  |  |  |  |  |  |  |  |  |
| - Applicants | *No outlier identified (see Table 1 for results)* | | | | | | | |  |  |  |  |  |  |  |  |  |  |
| - Non-contextualized | *No outlier identified (see Table 1 for results)* | | | | | | | |  |  |  |  |  |  |  |  |  |  |
| - Contextualized | *No outlier identified (see Table 1 for results)* | | | | | | | |  |  |  |  |  |  |  |  |  |  |
| Design |  |  |  |  |  |  |  |  |  |  |  |  |  |  |  |  |  |  |
| - Concurrent design | 104 | .16 | .14, .18 | .03, .28 | 197.65 | 47.89 | .074 | .15, .16; .16 | L | 17 | .13 | .11, .15 | .14 (.01) | .12 (.01) | .49 | .11 (.00) | .14 | .19 (.16,.22) |
| - Non-contextualized | 85 | .15 | .13, .17 | .02, .28 | 176.44 | 52.39 | .080 | .15, .15; .15 | L | 10 | .13 | .10, .15 | .13 (.01) | .10 (.01) | .49 | .11 (.00) | .13 | .20 (.17,.24) |
| - Contextualized | *No outlier identified (see Table 1 for results)* | | | | | | | |  |  |  |  |  |  |  |  |  |  |
| - Predictive design | *No outlier identified (see Table 1 for results)* | | | | | | | |  |  |  |  |  |  |  |  |  |  |
| - Non-contextualized | *No outlier identified (see Table 1 for results)* | | | | | | | |  |  |  |  |  |  |  |  |  |  |
| - Contextualized | *No outlier identified (see Table 1 for results)* | | | | | | | |  |  |  |  |  |  |  |  |  |  |
| Scale ^a^ |  |  |  |  |  |  |  |  |  |  |  |  |  |  |  |  |  |  |
| - NEO | 41 | .14 | .11, .18 | .02, .26 | 75.53 | 47.04 | .071 | .14, .15; .14 | L | 7 | .11 | .08, .15 | .13 (.00) | .10 (.01) | .57 | .11 (.01) | .13 | .19 (.14,.25) |
| - PCI | *No outlier identified (see Table 1 for results)* | | | | | | | |  |  |  |  |  |  |  |  |  |  |
| - PSI | *No outlier identified (see Table 1 for results)* | | | | | | | |  |  |  |  |  |  |  |  |  |  |

*Note:* *k* = number of correlation coefficients in the analyzed distribution. Publication bias analyses were not conducted for distributions with less than *k*=10; $\bar{r}_{o_{RE}}$ = random-effects weighted mean observed correlation; 95% CI = 95% confidence interval; 90% PI = 90% prediction interval; *Q* = weighted sum of squared deviations from the mean; *I*^2^ = ratio of true heterogeneity to total variation; *τ* = between-sample standard deviation; osr = one-sample removed, including the minimum and maximum effect size and the median weighted mean observed correlation; Trim and fill = trim and fill analysis; FPS = funnel plot side (i.e., side of the funnel plot where samples were imputed; L = left, R = right); *ik* = number of trim and fill imputed samples; t&f $\bar{r}_{o}$ = trim and fill adjusted observed mean (the weighted mean of the distribution of the combined observed and the imputed samples); t&f 95% CI = trim and fill adjusted 95% confidence interval; sm_m_ $\bar{r}_{o}$= one-tailed moderate selection model’s adjusted observed mean (and its variance); sm_s_ $\bar{r}_{o}$= one-tailed severe selection model’s adjusted observed mean (and its variance); Ex. sig. = excess significance; PET-PEESE = precision-effect test-precision effect estimate with standard error; PET $\bar{r}_{o}$ = PET adjusted observed mean (and its one-tailed *p*-value; PEESE $\bar{r}_{o}$ is the adjusted observed mean if PET $\bar{r}_{o}$ is significant, the PET $\bar{r}_{o}$ is the adjusted observed mean if the *p*-value is not significant [Stanley & Doucouliagos, 2014]); PEESE $\bar{r}_{o}$ = PEESE adjusted observed mean; P-TES = the probability of the chi-square test of excess significance; *p*-uniform (95% CI) = the *p*-uniform estimate and its 95% confidence interval; n/a = not applicable (because *k* was too small to conduct these analyses or because the variance component for the selection models indicated that the estimate was nonsensical; Kepes et al., 2012).

^a^ We only analyzed three scale distributions (i.e., NEO = NEO Personality Inventory, PCI = Personal Characteristics Inventory, and PSI = Personal Style Inventory) because the other distributions were too small to reach definite conclusions regarding the robustness of the meta-analytic mean estimate.
