# Supplementary material for: The Validity of Conscientiousness Is Overestimated in the Prediction of Job Performance
Source: PLoS One. 2015 Oct 30;10(10):e0141468. doi: 10.1371/journal.pone.0141468 (PMC4627756; doi:10.1371/journal.pone.0141468)
Supplement: S2 Table — Lowest value = lowest mean estimate from all analyses (r-oRE; osr, r-oFE, t&f r-o, smm r-o, sms r-o, and PET-PEESE; we did not include the p-uniform values due to the lack of convergence with the results of the other, more established methods; likely due to the poor performance of this method with heterogeneous data [van Assen et al., in press]); r-oRE = random-effects weighted mean observed correlation (the potentially best mean estimate); Highest value = highest mean estimate from all analyses (r-oRE; osr, r-oFE, t&f r-o, smm r-o, sms r-o, PET-PEESE); BRE = Baseline range estimate: the absolute range between r-oRE and the estimate farthest away (either the lowest or highest value); MRE = Maximum range estimate: the absolute range between the lowest or highest value. When calculating the relative difference of the range estimates, we used r-oRE, the potentially best mean estimate, as the base (i.e., as 100%). Ideally, BRE and MRE should be identical. If not, outliers or other artifacts may have caused such differences. Practical difference: negligible = if the relative range (BRE or MRE) is smaller than 20%; moderate = if the relative range (BRE or MRE) is larger than 20%; large = if the relative range (BRE or MRE) is larger than 40% [33]. (DOCX) [file pone.0141468.s002.docx]

**S2 Table. Robustness of results and conclusions of the analyses (outlier excluded)**

| Distribution | Lowest value | $\bar{r}_{o_{RE}}$ | Highest value | BRE | Practical difference | MRE | Practical difference | Conclusion ^a^ |
| --- | --- | --- | --- | --- | --- | --- | --- | --- |
| Conscientiousness | .12 ^f^ | .16 | .16 ^b, c^ | .04 (25%) | moderate | .04 (25%) | moderate | Moderate difference |
| Frame of reference |  |  |  |  |  |  |  |  |
| - Non-contextualized | .11 ^f^ | .15 | .16 ^c^ | .05 (31%) | moderate | .05 (31%) | moderate | Moderate difference |
| - Contextualized | *No outlier identified. See Table 2 for results and conclusions.* | | | | | | | |
| Source |  |  |  |  |  |  |  |  |
| - Journal articles | *No outlier identified. See Table 2 for results and conclusions.* | | | | | | | |
| - Non-contextualized | *No outlier identified. See Table 2 for results and conclusions.* | | | | | | | |
| - Contextualized | *No outlier identified. See Table 2 for results and conclusions.* | | | | | | | |
| - Non-journal articles | .07 ^f^ | .13 | .13 ^b, c, d, g^ | .06 (46%) | large | .06 (46%) | large | Large difference |
| - Non-contextualized | .04 ^f^ | .11 | .13 ^d, g^ | .07 (64%) | large | .09 (82%) | large | Large difference |
| - Contextualized | *No outlier identified. See Table 2 for results and conclusions.* | | | | | | | |
| Purpose |  |  |  |  |  |  |  |  |
| - General purpose | .09 ^f^ | .15 | .15 ^b, c^ | .06 (40%) | large | .06 (40%) | large | Large difference |
| - Non-contextualized | .08 ^f^ | .14 | .15 ^c^ | .06 (43%) | large | .07 (50%) | large | Large difference |
| - Contextualized |  | | | | | | | |
| - Workplace purpose | *No outlier identified. See Table 2 for results and conclusions.* | | | | | | | |
| - Non-contextualized | *No outlier identified. See Table 2 for results and conclusions.* | | | | | | | |
| - Contextualized | *No outlier identified. See Table 2 for results and conclusions.* | | | | | | | |
| Sample |  |  |  |  |  |  |  |  |
| - Incumbents | .12 ^f^ | .16 | .16 ^b, c^ | .04 (25%) | moderate | .04 (25%) | moderate | Moderate difference |
| - Non-contextualized | .10 ^f^ | .15 | .15 ^b, c^ | .05 (33%) | moderate | .05 (33%) | moderate | Moderate difference |
| - Contextualized | *No outlier identified. See Table 2 for results and conclusions.* | | | | | | | |
| - Applicants | *No outlier identified. See Table 2 for results and conclusions.* | | | | | | | |
| - Non-contextualized | *No outlier identified. See Table 2 for results and conclusions.* | | | | | | | |
| - Contextualized | *No outlier identified. See Table 2 for results and conclusions.* | | | | | | | |
| Design |  |  |  |  |  |  |  |  |
| - Concurrent design | .12 ^f^ | .16 | .16 ^b, c^ | .04 (25%) | moderate | .04 (25%) | moderate | Moderate difference |
| - Non-contextualized | .10 ^f^ | .15 | .15 ^b, c^ | .05 (33%) | moderate | .05 (33%) | moderate | Moderate difference |
| - Contextualized | *No outlier identified. See Table 2 for results and conclusions.* | | | | | | | |
| - Predictive design | *No outlier identified. See Table 2 for results and conclusions.* | | | | | | | |
| - Non-contextualized | *No outlier identified. See Table 2 for results and conclusions.* | | | | | | | |
| - Contextualized | *No outlier identified. See Table 2 for results and conclusions.* | | | | | | | |
| Scale |  |  |  |  |  |  |  |  |
| - NEO | .10 ^f^ | .14 | .15 ^c^ | .04 (29%) | moderate | .05 (36%) | moderate | Moderate difference |
| - PCI | *No outlier identified. See Table 2 for results and conclusions.* | | | | | | | |
| - PSI | *No outlier identified. See Table 2 for results and conclusions.* | | | | | | | |

*Note:* Lowest value = lowest mean estimate from all analyses ($\bar{r}_{o_{RE}}$; osr, $\bar{r}_{o_{FE}}$, t&f $\bar{r}_{o}$, sm_m_ $\bar{r}_{o}$, sm_s_ $\bar{r}_{o}$, and PET-PEESE; we did not include the *p*-uniform values due to the lack of convergence with the results of the other, more established methods; likely due to the poor performance of this method with heterogeneous data [van Assen et al., in press]); $\bar{r}_{o_{RE}}$ = random-effects weighted mean observed correlation (the potentially best mean estimate); Highest value = highest mean estimate from all analyses ($\bar{r}_{o_{RE}}$; osr, $\bar{r}_{o_{FE}}$, t&f $\bar{r}_{o}$, sm_m_ $\bar{r}_{o}$, sm_s_ $\bar{r}_{o}$, PET-PEESE); BRE = Baseline range estimate: the absolute range between $\bar{r}_{o_{RE}}$ and the estimate farthest away (either the lowest or highest value); MRE = Maximum range estimate: the absolute range between the lowest or highest value. When calculating the relative difference of the range estimates, we used $\bar{r}_{o_{RE}}$, the potentially best mean estimate, as the base (i.e., as 100%). Ideally, BRE and MRE should be identical. If not, outliers or other artifacts may have caused such differences. Practical difference: negligible = if the relative range (BRE or MRE) is smaller than 20%; moderate = if the relative range (BRE or MRE) is larger than 20%; large = if the relative range (BRE or MRE) is larger than 40% (Kepes et al., 2012).

^a^  Conclusions of a negligible difference indicate that the meta-analytic mean estimate (i.e., $\bar{r}_{o_{RE}}$) is likely to be robust. Conclusions of a moderate, moderate to large, or large difference indicates that the meta-analytic mean estimate (i.e., $\bar{r}_{o_{RE}}$) is likely to be non-robust and could be misestimated (i.e., $\bar{r}_{o_{RE}}$ could be under- or overestimated; typically overestimated in our analyses).

^b^ = value from $\bar{r}_{o_{RE}}$; ^c^ = value from osr, $\bar{r}_{o_{FE}}$; ^d^ = value from t&f $\bar{r}_{o}$; ^e^ = value from sm_m_ $\bar{r}_{o}$; ^f^ = value from sm_s_ $\bar{r}_{o}$; ^g^ = value from PET-PEESE (value from PEESE if the PET value was significant, value from PET if it was not significant).
