# Supplementary material for: The Validity of Conscientiousness Is Overestimated in the Prediction of Job Performance
Source: PLoS One. 2015 Oct 30;10(10):e0141468. doi: 10.1371/journal.pone.0141468 (PMC4627756; doi:10.1371/journal.pone.0141468)
Supplement: S3 Table — Lowest value = lowest mean estimate from all analyses (r-oRE; osr, r-oFE, t&f r-o, smm r-o, sms r-o, PET-PEESE, and p-uniform); r-oRE = random-effects weighted mean observed correlation (the potentially best mean estimate); Highest value = highest mean estimate from all analyses (r-oRE; osr, r-oFE, t&f r-o, smm r-o, sms r-o, PET-PEESE, and p-uniform); BRE = Baseline range estimate: the absolute range between r-oRE and the estimate farthest away (either the lowest or highest value); MRE = Maximum range estimate: the absolute range between the lowest or highest value. When calculating the relative difference of the range estimates, we used r-oRE, the potentially best mean estimate, as the base (i.e., as 100%). Ideally, BRE and MRE should be identical. If not, outliers or other artifacts may have caused such differences. Practical difference: negligible = if the relative range (BRE or MRE) is smaller than 20%; moderate = if the relative range (BRE or MRE) is larger than 20%; large = if the relative range (BRE or MRE) is larger than 40% [Kepes et al., 2012]. (DOCX) [file pone.0141468.s003.docx]

**S3 Table. Robustness of results and conclusions of the analyses (including *p*-uniform estimates)**

| Distribution | Lowest value | $\bar{r}_{o_{RE}}$ | Highest value | BRE | Practical difference | MRE | Practical difference | Conclusion ^a^ |
| --- | --- | --- | --- | --- | --- | --- | --- | --- |
| Conscientiousness | .12 ^f^ | .16 | .19 ^h^ | .04 (25%) | moderate | .07 (44%) | large | Moderate to large difference |
| Frame of reference |  |  |  |  |  |  |  |  |
| - Non-contextualized | .09 ^f^ | .15 | .20 ^h^ | .06 (40%) | large | .07 (47%) | large | Large difference |
| - Contextualized | .17 ^d, g^ | .19 | .20 ^c^ | .02 (11%) | negligible | .03 (16%) | negligible | Negligible difference |
| Source |  |  |  |  |  |  |  |  |
| - Journal articles | .07 ^g^ | .19 | .19 ^b, c^ | .12 (63%) | large | .12 (63%) | large | Large difference |
| - Non-contextualized | .07 ^g^ | .19 | .21 ^h^ | .12 (63%) | large | .14 (74%) | large | Large difference |
| - Contextualized | .07 ^g^ | .19 | .20 ^c^ | .12 (63%) | large | .13 (68%) | large | Large difference |
| - Non-journal articles | .10 ^e^ | .12 | .18 ^h^ | .02 (17%) | negligible | .08 (67%) | large | Negligible to large difference |
| - Non-contextualized | .08 ^e^ | .11 | .19 ^h^ | .03 (27%) | moderate | .11 (100%) | large | Moderate to large difference |
| - Contextualized | *Distribution is too small to reach definite conclusions regarding the robustness of the meta-analytic mean estimate* | | | | | | | |
| Purpose |  |  |  |  |  |  |  |  |
| - General purpose | .08 ^f^ | .14 | .20 ^h^ | .06 (43%) | large | .12 (86%) | large | Large difference |
| - Non-contextualized | .11 ^d, e^ | .14 | .15 ^c^ | .03 (21%) | moderate | .04 (71%) | large | Moderate to large difference |
| - Contextualized | *Distribution is too small to reach definite conclusions regarding the robustness of the meta-analytic mean estimate* | | | | | | | |
| - Workplace purpose | .16 ^g^ | .19 | .20 ^c^ | .03 (16%) | negligible | .04 (21%) | moderate | Negligible to moderate difference |
| - Non-contextualized | .09 ^g^ | .19 | .20 ^c^ | .10 (53%) | large | .11 (58%) | large | Large difference |
| - Contextualized | .19 ^c, d, e, f^ | .20 | .21 ^c^ | .01 (5%) | negligible | .02 (10%) | negligible | Negligible difference |
| Sample |  |  |  |  |  |  |  |  |
| - Incumbents | .11 ^f^ | .16 | .19 ^h^ | .05 (31%) | moderate | .08 (50%) | large | Moderate to large difference |
| - Non-contextualized | .09 ^f^ | .15 | .20 ^h^ | .06 (40%) | large | .11 (73%) | large | Large difference |
| - Contextualized | .11 ^g^ | .19 | .20 ^c^ | .08 (42%) | large | .09 (47%) | large | Large difference |
| - Applicants | *Distribution is too small to reach definite conclusions regarding the robustness of the meta-analytic mean estimate* | | | | | | | |
| - Non-contextualized | *Distribution is too small to reach definite conclusions regarding the robustness of the meta-analytic mean estimate* | | | | | | | |
| - Contextualized | *Distribution is too small to reach definite conclusions regarding the robustness of the meta-analytic mean estimate* | | | | | | | |
| Design |  |  |  |  |  |  |  |  |
| - Concurrent design | .11 ^f^ | .15 | .19 ^h^ | .04 (27%) | moderate | .05 (31%) | moderate | Moderate difference |
| - Non-contextualized | .09 ^f^ | .15 | .20 ^h^ | .06 (40%) | large | .06 (40%) | large | Large difference |
| - Contextualized | .11 ^g^ | .18 | .19 ^c^ | .07 (39%) | moderate | .08 (44%) | large | Moderate to large difference |
| - Predictive design | *Distribution is too small to reach definite conclusions regarding the robustness of the meta-analytic mean estimate* | | | | | | | |
| - Non-contextualized | *Distribution is too small to reach definite conclusions regarding the robustness of the meta-analytic mean estimate* | | | | | | | |
| - Contextualized | *Distribution is too small to reach definite conclusions regarding the robustness of the meta-analytic mean estimate* | | | | | | | |
| Scale |  |  |  |  |  |  |  |  |
| - NEO | .08 ^f, g^ | .14 | .19 ^h^ | .06 (43%) | large | .11 (79%) | large | Large difference |
| - PCI | .20 ^d^ | .24 | .25 ^c^ | .04 (17%) | negligible | .05 (21%) | moderate | Negligible to moderate difference |
| - PSI | .21 ^e, f^ | .22 | .22 ^b, c, d^ | .01 (5%) | negligible | .01 (5%) | negligible | Negligible difference |

*Note:* Lowest value = lowest mean estimate from all analyses ($\bar{r}_{o_{RE}}$; osr, $\bar{r}_{o_{FE}}$, t&f $\bar{r}_{o}$, sm_m_ $\bar{r}_{o}$, sm_s_ $\bar{r}_{o}$, PET-PEESE, and *p*-uniform); $\bar{r}_{o_{RE}}$ = random-effects weighted mean observed correlation (the potentially best mean estimate); Highest value = highest mean estimate from all analyses ($\bar{r}_{o_{RE}}$; osr, $\bar{r}_{o_{FE}}$, t&f $\bar{r}_{o}$, sm_m_ $\bar{r}_{o}$, sm_s_ $\bar{r}_{o}$, PET-PEESE, and *p*-uniform); BRE = Baseline range estimate: the absolute range between $\bar{r}_{o_{RE}}$ and the estimate farthest away (either the lowest or highest value); MRE = Maximum range estimate: the absolute range between the lowest or highest value. When calculating the relative difference of the range estimates, we used $\bar{r}_{o_{RE}}$, the potentially best mean estimate, as the base (i.e., as 100%). Ideally, BRE and MRE should be identical. If not, outliers or other artifacts may have caused such differences. Practical difference: negligible = if the relative range (BRE or MRE) is smaller than 20%; moderate = if the relative range (BRE or MRE) is larger than 20%; large = if the relative range (BRE or MRE) is larger than 40% (Kepes et al., 2012).

^a^  Conclusions of a negligible difference indicate that the meta-analytic mean estimate (i.e., $\bar{r}_{o_{RE}}$) is likely to be robust. Conclusions of a moderate, moderate to large, or large difference indicates that the meta-analytic mean estimate (i.e., $\bar{r}_{o_{RE}}$) is likely to be non-robust and could be misestimated (i.e., $\bar{r}_{o_{RE}}$ could be under- or overestimated; typically overestimated in our analyses).

^b^ = value from $\bar{r}_{o_{RE}}$; ^c^ = value from osr, $\bar{r}_{o_{FE}}$; ^d^ = value from t&f $\bar{r}_{o}$; ^e^ = value from sm_m_ $\bar{r}_{o}$; ^f^ = value from sm_s_ $\bar{r}_{o}$; ^g^ = value from PET-PEESE (value from PEESE if the PET value was significant, value from PET if it was not significant); ^h^ = value from *p*-uniform.
