# Supplementary material for: The Validity of Conscientiousness Is Overestimated in the Prediction of Job Performance
Source: PLoS One. 2015 Oct 30;10(10):e0141468. doi: 10.1371/journal.pone.0141468 (PMC4627756; doi:10.1371/journal.pone.0141468)
Supplement: S4 Table — (DOCX) [file pone.0141468.s004.docx]

**S4 Table. Moderator statistical tests using between-group *Q* test (outlier excluded)**

| Distribution | Between-group *Q* | *p*-value |
| --- | --- | --- |
| Frame of Reference: Non-contextualized vs. Contextualized | 3.00 | .08 |
| Source: Journal articles vs. non-journal articles | 8.48 | .00 |
| - Journal articles: Non-contextualized vs. Contextualized | *No outlier. See Table 3.* | |
| - Non-Journal articles: Non-contextualized vs. Contextualized | 6.73 | .01 |
| Purpose: General vs Workplace | 6.05 | .01 |
| - Purpose: General: Non-contextualized vs. Contextualized | .66 | .42 |
| - Purpose: Workplace: Non-contextualized vs. Contextualized | *No outlier. See Table 3.* | |
| Sample: Incumbents vs. Applicants | 4.21 | .04 |
| - Sample: Incumbents: Non-contextualized vs. Contextualized | 2.80 | .09 |
| - Sample: Applicants: Non-contextualized vs. Contextualized | *No outlier. See Table 3.* | |
| Design: Concurrent vs. Predictive | 8.04 | .01 |
| - Design: Concurrent: Non-contextualized vs. Contextualized | 1.98 | .16 |
| - Design: Predictive: Non-contextualized vs. Contextualized | *No outlier. See Table 3.* | |
| Scale: NEO vs. PCI vs. PSI | 13.64 | .00 |
| - NEO vs. PCI | 11.46 | .00 |
| - NEO vs. PSI | 6.28 | .01 |
| - PCI vs. PSI | *No outlier. See Table 3.* | |
